# A fully human afucosylated anti-GARP antibody PBA-0111 drives anti-tumor immunity through combined ADCC induction and TGF-β1 pathway blockade

**DOI:** 10.3389/fimmu.2026.1921265

**Published:** 2026-09-17

**Authors:** Pieter Spee, Monika Litwin, Damian Trojanowski, Connor Burns, Adrianna Dudek, Elżbieta Gołdyn, Kamilla Sołtys, Michał Sobecki, Aleksandra Świderska, Irwin Matyjaszczyk, Marek Orłowski, Piotr Mamczur, Robert Kolodziejczyk, Dmytro Piliuhin, Jiaming Xu, Ewelina Krzywińska, Sameer Deshmukh, Tomasz Klaus, Ewelina Świderek, Kate Gillespie, Atticus Turner, Wendy Jenkins, Jason Frazier, Kenneth R. Gundle, John Weinberg, Niina Veitonmäki

**Affiliations:** 1 MultiQure S.A (Formely Pure Biologics), Wrocław, Poland; 2 Presage Biosciences, Inc., Seattle, WA, United States; 3 Department of Biochemistry, Molecular Biology and Biotechnology, Faculty of Chemistry, Wrocław University of Science and Technology, Wrocław, Poland; 4 GemPharmatech, Jiangsu, China; 5 Oregon Health and Sciences University, Portland, OR, United States

**Keywords:** GARP, immuno-oncology, immunotherapy, PBA-0111, Phase 0, soft tissue sarcoma, TGF-β1, Tregs

## Abstract

**Methods:**

We have evaluated PBA-0111, a fully human antibody targeting Glycoprotein A Repetitions Predominant (GARP), designed to induce both antibody-dependent cellular cytotoxicity (ADCC) as well as blockage of Transforming Growth Factor Beta 1 (TGF-β1) activation. Functional assessments included *in vitro* cytotoxicity assays, inhibition of GARP/TGF-β1 complex formation and inhibition of TGF-β1 signaling. PBA-0111 was compared with clinically tested anti-GARP antibodies, including DS-1055a- and ABBV-151-like antibodies. Lack of significant binding to human platelets was confirmed by flow cytometry. Anti-tumor efficacy *in vivo* was assessed in CD34^+^ human stem cell humanized mice xenograft models, including models where GARP was expressed both on tumor cells and regulatory T cells as well as models where GARP was expressed exclusively on regulatory T cells. The potential of PBA-0111 to stimulate immune cell activation inside tumors was further studied in a placebo-controlled Phase 0 clinical study in soft tissue sarcoma patients receiving intra-tumoral injections.

**Results and discussion:**

Our findings demonstrate that PBA-0111 exhibits ADCC activity comparable to DS-1055a-like antibody, which was not observed for ABBV-151-like antibody. PBA-0111 effectively blocks TGF-β1 activation, similar to ABBV-151-like antibody but unlike DS-1055a-like antibody. In contrast to ABBV-151, which blocks TGF-β1 activation within the pre-formed GARP/TGF-β1 complex, PBA-0111 prevents GARP/TGF-β1 complex formation itself. Importantly, PBA-0111 did not demonstrate significant binding to human platelets. In humanized mice, whether GARP was expressed on tumor cells or exclusively on Tregs, PBA-0111 treatment induced robust inhibition of tumor growth, whereas DS-1055a-like antibody did not demonstrate any statistically significant anti-tumor efficacy in these models. Tumor growth inhibition by PBA-0111 was accompanied by cytotoxic T cell activation. A similar trend of T cell activation was observed in soft tissue sarcoma patients receiving intra-tumoral injections of PBA-0111 in the Phase 0 clinical trial.

**Conclusion:**

These results underscore the therapeutic potential of PBA-0111 as a dual-functioning immunotherapy and a promising candidate for the treatment of GARP-positive cancers. This integrated mechanism may amplify anti-tumor immunity, improve synergy with checkpoint inhibitors, and provide more durable reprogramming of the tumor microenvironment than either approach alone.

## Introduction

Effective cancer immunotherapy requires two complementary actions: increasing tumor cell visibility to the immune system and triggering cytotoxic responses, while simultaneously eliminating the immunosuppressive environment that tumors actively create to evade destruction. A central challenge is the tumor microenvironment (TME), which develops a strongly immunosuppressive milieu that promotes tumor growth and progression by impairing the immune system’s ability to recognize and eliminate malignant cells, - a mechanism widely recognized as a hallmark of cancer (1, 2).

Among the key mediators of immune regulation in cancer is Transforming Growth Factor-β1 (TGF-β1), a cytokine with central roles in maintaining immune tolerance and physiological homeostasis. In the context of cancer, TGF-β1 is often abundantly present in the TME, where tumors exploit its signaling pathway to suppress anti-tumor immunity, particularly in advanced stages of the disease. Elevated TGF-β1 activity has been associated with resistance to immune checkpoint blockade in several malignancies, including triple-negative breast cancer, metastatic colorectal cancer, and urothelial carcinoma (3–6). Despite its importance as a therapeutic target, direct inhibition of TGF-β1 signaling remains challenging and can lead to adverse effects such as inflammatory, autoimmune, or cardiovascular complications or even paradoxical tumor promotion (7–11).

An emerging strategy to modulate TGF-β1 activity more selectively involves targeting its upstream regulatory mechanisms. Glycoprotein-A repetitions predominant (GARP), encoded by *LRRC32*, functions as a cell-surface receptor that binds latent TGF-β and facilitates its activation. GARP can present all three latent TGF-β isoforms (LTGF-β1, LTGF-β2, and LTGF-β3) and is expressed on multiple cell types within the TME, including platelets, activated regulatory T cells (Tregs) and B cells, some mesenchymal stromal cells, endothelial cells, fibroblasts and certain cancer cells (12–18). GARP exists in several forms, including ligand-free membrane-bound GARP, membrane-bound GARP/TGF-β1 complex and soluble GARP generated through proteolytic shedding (17, 19). These distinct forms are differentially expressed across cell types. Notably, Tregs and tumor cells express both ligand-free and complexed GARP, whereas platelets display GARP in complex with latent TGF-β1 (12, 20). This heterogeneity contributes to the complexity of GARP-dependent TGF-β1 signaling and enhances its immunosuppressive potential.

Therapeutic strategies that disrupt GARP/TGF-β1 interactions have shown promise in reducing tissue fibrosis and restoring anti-tumor immune responses. Monoclonal antibodies such as livmoniplimab (ABBV-151) bind GARP/latent TGF-β1 complex, preventing integrin-mediated activation of TGF-β1 leading to enhanced CD8^+^ T cell-mediated anti-tumor responses, including improved anti-tumor efficacy of anti-PD-1 therapy (21, 22). Similar strategies have been pursued with HLX60, JYB1907 and BPB-101 antibodies (23–25). Beyond blocking activation of the pre-formed complex, preventing GARP/latent TGF-β1 complex formation itself represents an additional therapeutic avenue, as described for PIIO-1 antibody (20). Since activated regulatory T cells can display both GARP and GARP/TGF-β1 on their cell surface, antibody targeting free GARP could intercept latent TGF-β1 before complex formation occurs, preventing surface display and thereby preventing immunosuppressive TGF-β1 activation. A completely different strategy involves depletion of GARP expressing Tregs, exemplified by DS-1055a, which targets both GARP and GARP/TGF-β1 complex to reduce immunosuppression by depleting Tregs (26). Thus, there are two opposing classes of GARP-targeting therapeutic antibodies that aim to overcome immune-suppression: 1) antibodies that functionally block GARP, but that do not attract immune cells to induce ADCC, and 2) antibodies that do not functionally block GARP, but that attract immune cells to induce ADCC towards GARP-expressing cells. Importantly, all these strategies share a key advantage over pan-TGF-β inhibition: context-selectivity, restricting intervention to TGF-β1 produced by GARP expressing cells while preserving the systemic TGF-β1 biology required for immune homeostasis and tissue maintenance. Yet, next-generation immunotherapy strategies could combine the two modes of action, within one single molecule.

Here we introduce PBA-0111, a novel fully human anti-GARP and anti-GARP/TGF-β1 complex antibody in afucosylated IgG1 designed to combine two complementary mechanisms: 1) inhibition of TGF-β1 activation, and 2) antibody-dependent cellular cytotoxicity (ADCC)-mediated depletion of GARP-expressing Tregs and tumor cells, benchmarked against in-house surrogate antibodies representing ABBV-151 and DS-1055a (**Supplementary Table 1**). We describe the development, characterization, preclinical- and early clinical evaluation of PBA-0111, a potential first-in-class afucosylated antibody targeting both GARP and GARP/TGF-β1 complex, with dual-mechanism activity, witch has recently completed a Phase 0 clinical study (NCT06700070) in patients with soft tissue sarcoma.

## Materials and methods

### Cell lines and cell line generation

HEK293T (CRL-3216), Raji (CCL-86) and Jurkat (clone E6-1, TIB-152) cells were obtained from ATCC. L-428 Hodgkin lymphoma cells were obtained from DSMZ (ACC 197). HEK29T cells were cultured in high glucose DMEM (Gibco™, 11960-044) supplemented with 10% Fetal Bovine Serum (FBS, Sigma Aldrich, F9665-500ML) and L-glutamine (Biowest, X0551-100). All other cells were cultured in RPMI 1640 medium (Gibco™, 31870074) supplemented with 10% Fetal Bovine Serum (FBS, Sigma Aldrich, F9665-500ML, (except 20% for L-428)), 1% GlutaMax (Gibco™, 35050061) and 1% Penicillin/Streptomycin Solution (Gibco™, 15140130). Cells were maintained at 37 °C, 5% CO2 and all experiments were performed using cells with fewer than 20 passages.

Stably transfected HEK293T and Raji cell lines were generated via electroporation (Gene Pulser, 200 V, 950 µF; single pulse) using the Sleeping Beauty Transposase system, with either empty vector, GARP alone, or GARP together with latent TGF-β1 complex. Jurkat cells were similarly transfected with plasmids encoding Integrin-beta8 (ITGβ8) and GARP. Stable populations were selected and maintained under continuous selection pressure using hygromycin and/or puromycin.

### Antibody generation

Phage display selections were conducted using a fully human single-chain variable fragment (scFv) Hyperimmune Library (Twist Biosciences, USA) against HEK293T cells expressing GARP/TGF-β1. Monoclonal phages were isolated from enriched pools and sequenced by Sanger sequencing (LCG, Germany). Unique clones were screened as phages by ELISA and flow cytometry for binding and specificity. The top clones were expressed as human IgG1 in CHO-KI cells (BioIntron, China) and evaluated for binding, affinity, specificity, and ADCC induction. Selected clones were further expressed as afucosylated IgG1 in GlyMaxX CHO cells (Evitria, Switzerland). Clinical drug substance was produced using FUT8 knock-out cells at WuXi Biologics (China). The sequence of PBA-0111 is described in the patent: WO2024251839/A1.

Reference antibodies ABBV-151- (both as MHG-8 and LHG10.6 clones), DS-1055a- and ADWA11-like (anti-avβ8) antibodies were generated based on publicly available sequences (26–30). Unless otherwise specified, ABBV-151-like antibody refers to clone LHG10.6. MHG-8 was also generated as IgG1 format as a control. Isotype controls were generated via phage display against streptavidin, and the selected antibodies were expressed in both native and afucosylated IgG1 formats (31). The control antibodies are referred to as Ctr IgG1 and IgG1.aF, respectively.

### Recombinant protein production

Recombinant His-tagged GARP extracellular domain (ECD), GARP/TGF-β1 complex, latent TGF-β1, and active TGF-β1 were generated in-house using Expi-CHO-S or ExpiHEK293 expression systems. Proteins were purified by immobilized metal affinity chromatography (IMAC), with double purification rounds applied where required to achieve sufficient purity. Identity and quality were assessed by SDS-PAGE, SEC-HPLC, differential scanning fluorimetry (Tycho), and PNGaseF deglycosylation, with all preparations benchmarked against commercially available reference proteins. Concentrations were determined by UV absorbance and densitometric analysis. Freeze-thaw stability was confirmed by a minimum of two cycles, and purified proteins were stored in pre-tested formulations at -80 °C until use.

### Cell-based binding assays

Cells overexpressing GARP, GARP/TGF-β1 or empty vector control, as well as cells with endogenous GARP/TGF-β1 expression, were labeled with CFSE CellTrace™ (Invitrogen, C34554), at room temperature (RT), for 30 min. Following washing, cells were incubated with serial concentrations of anti-GARP antibodies (PBA-0111, DS-1055a-like or ABBV-151-like antibody) for 30 min on ice. Human LAP (TGF-beta 1) Alexa Fluor^®^ 647 (R&D Systems, IC77541R) and Human LRRC32/GARP Alexa Fluor^®^ 647 (R&D Systems, FAB6055R) were used as positive controls. Cells were subsequently stained with APC-conjugated goat anti-human IgG Fcγ-specific secondary antibody (Jackson ImmunoResearch, 109-005-008), followed by Sytox Blue (Invitrogen, S34857). Samples were analyzed using an Attune™ NxT flow cytometer (Thermo Fisher Scientific) and analyzed using FlowJo X v.10.8.1.

### Molecular binding assays using SPR

The binding kinetics were measured using the Biacore 8K instrument (Cytiva). Anti human IgG (Fc) antibodies (Cytiva, 29234600) were immobilized on a CM5 chip by direct amine coupling to capture tested antibodies. For GARP binding, PBA-0111, DS-1055a-like IgG1 and ABBV-151-like IgG4 (0.1µg/mL) were captured, followed by injection of serial dilution of human GARP or GARP-complex (1500-23.4 nM and 500-7.81 nM). Data were double referenced and analyzed using a 1:1 Langmuir model to calculate *k_on_, k_off_*, and *K_D_.*

The antibody affinities to Fcγ receptors were determined with a Biacore 8K instrument (Cytiva). Recombinant biotinylated FcγR proteins (Sino Biological, #10389-H27H-B, #10389-H27H1-B) were immobilized on the CAP chip (Cytiva), according to the supplier’s instruction. Antibodies were injected using serial dilutions, and kinetic parameters were determined as described above.

### Interaction with GARP/TGF-β1 subdomains and specificity assessment using BLI

Binding to GARP/TGF-β1 subdomains and specificity was assessed using an Octet RED384 (Sartorius). Antibodies were immobilized on AHC (Anti-hIgG Fc Capture) biosensors (Sartorius) at 15 μg/mL. Recombinant subdomain proteins: GARP/TGF-β1, GARP, LAP-TGF-β1 (Sigma-Aldrich, L3408), latent TGF-β1/His, TGF-β1, latent TGF-β2/His (Acro Biosystems, TG2-H5243), latent TGFβ3/His (AcroBiosystems, TG3-H52H5) and LRRC15/His (Acro Biosystems, LR5-H52H3) at 500 nM were used as analytes. Association and dissociation phases were recorded, and data were analyzed using Octet Analysis Studio12.2 software (Sartorius).

### GARP/TGF-β1 complex blocking assay

Raji-GARP cells were incubated with PBA-0111, DS-1055-like IgG1, ABBV-151-like IgG4 or isotype control IgG1.aF at 0.2 ng/mL-50 µg/mL (1,4 pM-0,3 µM), prior to or together adding latent TGF-β1 (1.5 µg/mL). After incubation (37 °C, 60 min), the cells were stained with anti-LAP-AF647 antibody (R&D Systems, IC77541R), followed by Sytox Blue (Invitrogen, S4857). Anti-LRRC32-AF647 (R&D Systems, FAB6055R) and anti-LAP-AF647 were used as staining controls. Samples were acquired using Attune NxT Flow Cytometer equipped with CytKick Max autosampler and analyzed using FlowJo X v.10.8.1.

### TGF-β1 activation assay (pSMAD detection)

SMAD2 phosphorylation was assessed by western blot as previously described (22). Jurkat wild-type and Jurkat-β8/GARP cells were stimulated with coated anti-CD3 (OCT3 clone, BioLegend, 317326, 1 μg/mL) and soluble anti-CD28 (BD Biosciences, 556620, 1 μg/mL) in the presence of ABBV-151-like IgG4, PBA-0111, DS-1055-like IgG1, IgG1 controls or ADWA11-like monoclonal anti-β8 antibody at 20 μg/mL. Cell lysates were collected after 24 h to ice-cold Sodium Borate Buffer supplemented with proteases (Complete Protease Inhibitor Cocktail, Roche, 4693116001) and phosphatase (PhosSTOP, Roche, 4906837001) inhibitors. Protein lysates were run in 4-20% Mini-Protean TGX Gel (BioRad), transferred to membranes followed by overnight incubation with anti-pSMAD2 antibody (Cell signalling technology, 3108L) and subsequent anti-rabbit IgG-HRP (R&D Systems, AF008) for one hour at RT. Detection was performed with Immobilon Western Chemiluminescent HRP Substrate using ChemiDoc MP (BioRad). Anti-GAPDH antibody (Invitrogen, MA5-15738-HRP) was used for loading control.

### PBMC and immune cell isolations

Peripheral blood mononuclear cells (PBMCs) were isolated from healthy human buffy coats obtained in an agreement with the local blood donation center in Wrocław. Isolation was performed using density gradient centrifugation (SepMate™-50 PBMC Isolation Tubes and Lymphoprep, Stemcell™, 85460 and 07811). Residual erythrocytes were lyzed with Red Blood Cell Lysis Buffer (BioLegend, 420302).

Platelet-rich plasma was collected from the above supernatant and processed separately. Platelets were washed and resuspended in Tyrode’s solution (137 mM NaCl, 2.7 mM KCl, 1 mM MgCl_2_, 1.8 mM CaCl_2_, 0.2 mM Na_2_HPO_4_,12 mM NaHCO_3_, 5.5 mM D-glucose) at a concentration of 25×10^6^ cells per mL.

Regulatory T cells were isolated from PBMCs from the same donor as NK cells by immunomagnetic two-step procedure with human CD4^+^CD25^+^ Regulatory T Cell Isolation Kit (Miltenyi Biotec, 130-091-301) according to the manufacturer’s protocol. Treg were activated using Dynabeads™ Human T-Activator CD3/CD28 for T Cell Expansion and Activation (Gibco, 11161D) for two days. Isolation purity and phenotyping of Tregs was performed using LIVE/DEAD™ Fixable Aqua Dead Cell Stain Kit (Invitrogen™, L34966), anti-CD4-PerCP/Cyanine5.5, (Biolegend, 300529), anti-hCD25-BV605 (BD Horizon, 562660), anti-hCD45-Brilliant Violet 785™ (BioLegend, 304048), anti-CD127-BV650 (BD Horizon, 63225), anti-GARP-PE/Cy7 (BioLegend, 352508), and anti-FoxP3-Alexa Fluor^®^ 647 (BD Pharmingen, 560045) antibodies.

### Platelet binding

Platelets were incubated with biotinylated PBA-0111, DS-1055a-like, ABBV-151-like and IgG1.aF antibodies at 10 µg/mL, followed by viability marker (LIVE/Dead™ Fixable Violet - LIVE/Dead™ 405 nm Stain, Thermo Fisher Scientific, L34955), and lineage markers; anti-CD41 (BioLegend, Clone HIP8, 303725), anti-CD42b (BioLegend, Clone HIP1, 303920), anti-CD45 (BioLegend, Clone HI30, 563716), and subsequently with FITC Streptavidin (BioLegend, 405202). Stained cells were acquired on a Cytek Flow Cytometer and analysis was done using FlowJo X v.10.0. Results are presented as mean values and standard deviation of fold change of FITC MFI.

### ADCC

Natural Killer (NK) cells were isolated from PBMCs by negative selection with NK Cell Isolation Kit (Miltenyi Biotec, 30-092-657) according to the manufacturer’s protocol. NK cells were characterized by purity and phenotype. Target cells were labeled with 0.1 µM of CellTrace™ CFSE Proliferation Kit (Invitrogen™, C34554) and pre-incubated with serial dilutions of tested antibodies. NK cells were added at an effector-to-target ratio of 10:1. After four hours of incubation at 37 °C, cells were stained with Live/Dead™ Fixable Violet Dead CellStain Kit solution (Invitrogen™), fixed with 4% paraformaldehyde (Thermo Scientific™), and analyzed by flow cytometry. Data was analyzed using FlowJoTM v10.8.1 Software (BD Life Sciences) and GraphPad Prism 7.05 software (GraphPad Software, Inc).

### NK cell activation

Isolated PBMCs were pre-activated overnight with 1 ng/mL human IL-15 (Biolegend, 570306). Target cells were preincubated with 50 nM of either PBA-0111, ABBV-151-like, DS-1055a-like antibodies or afucosylated isotype control. Subsequently, washed target cells were incubated with PBMC for 4 hours at 37 °C (E:T 1:1), in the presence of APC-conjugated human CD107a antibody (BD, 560664) and protein transport inhibitors (BD GolgiStop, 554724 and BD GolgiPlug, 555029). Subsequently, cells were incubated with blocking solution, then labelled with Live/Dead™ Fixable Aqua Dead CellStain Kit solution (Invitrogen™), BV605 anti-human CD56 (BD Biosciences, Clone: NCAM16.2, 562779), BV421 anti-human CD16 (Biolegend, Clone: 3G8, 302038), APC-H7 anti-human CD3 (BD Biosciences, Clone: SK7, 560176), fixed with BD Cytofix/Cytoperm™ (BD Biosciences, 554714), followed by intracellular staining for BB700 anti-human IFN-γ (BD Biosciences, Clone: B27, 566394) and BV750 anti-human TNF-α (BD Biosciences, Clone: Mab11, 566359). Isotype-matched antibodies from the same manufacturer (BD Biosciences or Biolegend) were used to assess background fluorescence. Cells were acquired with Cytek^®^ flow cytometer (Cytek Biosciences) and data were analyzed using FlowJo v10. Results are expressed as the percentage of CD107a, IFN-γ and TNF-α-positive cells to the total of CD56-positive CD3-negative cells according to gating strategy (**Supplementary Figure 7**).

### *In vivo* anti-tumor efficacy studies

#### huHSC-NSG-hIL15 Raji-GARP/TGF-β1 model

The human hematopoietic stem-cell NOD scid gamma- hIL15 (huHSC-NSG-hIL15) Raji-GARP/TGF-β1 model was performed at Jackson Laboratories (Sacramento, CA, US), an AAALAC-accredited institution. All procedures were reviewed and approved by The Jackson Laboratory Institutional Animal Care and Use Committee (IACUC) and conducted in accordance with the *Guide for the Care and Use of Laboratory Animals* and applicable federal regulations.

Humanized NSG-hIL15 mice reconstituted with human hematopoietic stem cells (huHSC-NSG-hIL15), aged 21–22 weeks, were used for tumor implantation. Raji cells stably overexpressing GARP/TGF-β1 (2 × 10^6^ cells in Matrigel) were inoculated subcutaneously into the upper flank of each mouse. During tumor inoculation, anesthesia was induced and maintained with isoflurane (2-3%) vaporized in 100% oxygen delivered at 1 L/min. Tumor growth was monitored regularly using caliper measurements, and tumor volume was calculated using the standard formula: (length × width²)/2. Once tumors reached a volume of 80-150 mm^3^, animals were randomly divided into treatment groups (n= 6-7/group) based on stem cell donor, tumor volume and mouse weight. Mice were treated intravenously with vehicle, PBA-0111, or DS-1055a-like antibody at 10 mg/kg. Treatments were administered twice weekly for a total of six doses. The tumor volume was measured twice weekly, and mice were monitored daily. At the end of the study, mice were euthanized by CO_2_ asphyxiation using gradual displacement of chamber air (30-70% chamber volume/min) in accordance with the AVMA Guidelines for the Euthanasia of Animals. Euthanasia was confirmed by cervical dislocation. At termination peripheral blood, tumors and spleens were collected. Tumors and spleens were excised, weighed, and divided into portions. One portion was mechanically and/or enzymatically processed into a single cell suspension. Terminal blood samples were subjected to red blood lysis.

Single-cell suspensions derived from tumors, spleens, and blood were stained with fluorescently labeled antibodies for flow cytometric analysis. The staining panel included markers for viability and human immune populations, including Live/Dead blue-UV-466A, hCD45-Fitc, hCD3-BV510, hCD8-APC-fire 750, hCD16-UV395, hCD56-BV605, hCD25-BV711, hFOXP3-PE, hCD69-BV421, and hCD19-APC. Counting beads were used as controls. Flow cytometry analysis was performed with BD FACS Diva 9.6.

#### huHSC-NCG-hIL15 HT-29 model

HT-29 colon carcinoma model was performed at GemPharmatech (Nanijang, China) in accordance with local ethical regulations and institutional guidelines for animal care and use. Female huHSC-NCG-hIL15 mice (GemPharmatech) were established by reconstitution of CD34^+^ huHSC from independent donors into irradiated NCG-hIL15 recipient mice and reconstitution level was confirmed by flow cytometry as >25% hCD45^+^ cells among total viable peripheral blood leukocytes. HT-29 tumor cells in DPBS were injected subcutaneously into right flank. No anesthesia was used during tumor inoculation. When the mean tumor volume reached approximately 80 mm^3^, mice were randomized into treatment groups based on the stem cell donor, humanization levels, mouse weight and tumor volumes. Mice received intravenous injections of either vehicle, PBA-0111, or DS-1055a-like antibody at a dose of 10 mg/kg. Treatments were given twice weekly. Tumor size was measured twice weekly, and blood samples were collected via retro-orbital bleeding at 4 and 24 hours following the first antibody treatment. Animals were monitored daily.

At the end of the study, mice were euthanized by CO_2_ asphyxiation using gradual displacement of chamber air (30-70% chamber volume/min). Euthanasia was confirmed by cervical dislocation. At termination tumors and spleens were collected, weighed, and divided into two portions. One portion of each tissue was processed into single cell suspension. After cell counting, an aliquot of cells was used for flow cytometry analysis. Terminal blood samples were subjected to red blood cell lysis prior to staining. Cells were stained with a panel of fluorescently labeled antibodies to assess immune cell composition and activation status. The panel included:Bioscience™ Fixable Viability Dye eFluor™ 506, hCD45-PerCP-Cy5.5, hCD3-AF700 (Biolegend, 300424), hCD4-APC- CY7 (BD, 557871), CD8-BV650 (BD, 563821), CD25-BV711 (BD, 563159), hCD127-AF647 (BD, 558598) hCD56-PE (BioLegend, 362508), hCD69-PED594 (Biologend, 310942), hCD107a-PE-CY-7 (Biolegend, 328618), CD16-BC785 (Biolegend, 302046), hGARP-BV421 (BD, 563956) and hCD134-BV-605 (Biolegend, 320832). Flow cytometry data were acquired and analyzed to quantify immune cell populations. To account for differences in tissue size, the number of analyzed cells was normalized to 0.1 g of the original tumor or spleen tissue weight. Fluorescence minus one (FMO) samples were used to set up the gates. The gating strategy used for analysis is provided in **Supplementary Figure 8**.

The remaining portions of the tumor tissues were fixed with 4% paraformaldehyde (PFA) and processed for immunohistochemical (IHC) analysis. Tumor sections were stained for GARP (LS Bio, LS-C98385-400), CD4, CD8, FoxP3, CC3, CD56, Granzyme-B, (Cell Signaling technology, 48274, 85336, 98377, 9664S, 99746, 46890, respectively), CD16 (Protein 245 Tech,16559-1-AP), IFN-γ (Abcam, 231036), and secondary antibodies from AKOYA (OP-001001, OP-001004, OP-001006, and OP-001006). Stained slides were imaged and analyzed using Vectra M3 3D 246 Imaging system (3D). Images were quantified as number of positive cells, % positive cells of DAPI positive cells and H-scores.

### Phase 0 clinical trial in soft tissue sarcoma patients

Patients were enrolled in an Institutional Review Board (IRB)-approved (IRB00000533), unblinded, open-label, multi-center clinical trial following written informed consent (NCT06700070). The study was conducted in accordance with the Declaration of Helsinki and International Council for Harmonisation of Technical Requirements for Pharmaceuticals for Human Use Good Clinical Practice guidelines. Eligible participants were adults (≥18 years) with histologically confirmed surgically resectable soft tissue sarcoma tumors that were accessible for intratumoral injections in patients scheduled for standard-of-care resection. All patients were recruited to the site in Oregon Health and Science University. Patient characteristics can be found in **Supplementary Table 5**.

Patients were injected with a total of 149.4 µg of PBA-0111 (24.9 µg in 8.3 µL/needle, 6 needles, and 2 vehicle needles) using the Comparative *In Vivo* Oncology (CIVO^®^) platform consisting of a single-use handheld microdose injector (MID) and fluorescent tracking microspheres (CIVO GLO Red and Yellow; ~4.5 μm inert polystyrene beads). The MID includes a transfer vessel for loading up to eight agents and a detachable injector configurable with 3-8 25-gauge needles. Adjustable needle depth and retraction enable controlled deposition of linear microdose columns (≤10 mm length) within tumors up to 3 cm deep. Based on prior diffusion estimates (~1.5 mm radius), each injection generates discrete drug-exposed regions suitable for spatial pharmacodynamic analysis (**Supplementary Figure 9**).

Treated tumors were resected 2-96 h post-injection according to the protocol, based on the planned tumor resection schedule. Injected regions were identified using fluorescence visualization of CIVO GLO microspheres and sectioned perpendicular to injection tracks (~4 mm intervals). Tissue samples were fixed in 10% buffered formalin supplemented with phosphatase inhibitors, followed by formalin fixation and paraffin embedding (FFPE).

### Histology and spatial biomarker analysis

Formalin-Fixed Paraffin-Embedded (FFPE) tissue sections (4 μm) were analyzed using hematoxylin and eosin staining, immunohistochemistry (IHC), and *in situ* hybridization (ISH). For fluorescent IHC, sections underwent antigen retrieval, blocking, and incubation with primary antibodies followed by fluorophore- or horseradish peroxidase-conjugated secondary antibodies with Opal-based detection. Slides were counterstained with DAPI (**Supplementary Material and Methods**).

ISH was performed using RNAscope multiplex fluorescent assays. Whole-slide imaging was conducted using automated digital microscopy platforms, enabling spatially resolved assessment of tumor and immune responses at defined sites of drug exposure (**Supplementary Material and Methods**).

### Statistics

The statistics were calculated using GraphPad Prism version 7.05 or 9 Kruskal-Wallis with Dunn´s multiple comparisons tests. For single comparisons Mann-Whitney non-parametric analysis was performed. Tumor growth curves were considered statistically significant using 2-way ANOVA, with Bonferroni multiple comparisons. All statistics are two-tailed p value.

## Results

### PBA-0111 antibody binds GARP independently of TGF-β1

Using phage display technology, a fully human GARP/GARP-TGF-β1-specific IgG1 monoclonal antibody, named PBA-0111, was generated capable of both killing GARP-expressing tumor cells and Tregs as well as blocking GARP-mediated TGF-β1 activation. The specificity of PBA-0111 for GARP was confirmed using a combination of molecular and cellular binding assays.

Using HEK293T cells transfected with cDNA encoding either human *GARP, GARP/TGF-β1* or empty vector, PBA-0111 bound to HEK293T-GARP cells with a half maximal effective concentration (EC50) of 0.74 nM and to GARP/TGF-β1 with an EC50 of 0.6 nM (**Figure 1A**; **Table 1**, n=3 independent experiments in duplicates) but showed no binding to empty vector-transfected cells. For comparison, anti-GARP mAb clone LHG10.6 (original Lama clone), mimicking ABBV-151, a humanized IgG4 mAb tested in various clinical trials, showed no binding to HEK293T-GARP cells and a binding EC50 of 0.48 nM to HEK293T-GARP/TGF-β1 cells (**Table 1**; **Supplementary Figure 1A**). The DS-1055a-like fully human IgG1 antibody, which has completed phase 1 clinical trials, bound both HEK293T-GARP and GARP/TGF-β1 cells with EC50 values of 0.35 nM and 0.42 nM, respectively (**Table 1**; **Supplementary Figure 1B**). The binding of PBA-0111 to endogenous GARP/TGF-β1 was confirmed using human cancer cells, including the Hodgkin lymphoma cell line L-428, with a binding EC50 of 0.53 nM. For comparison, ABBV-151-like and DS-1055a-like antibodies showed average EC50 values for binding GARP/TGF-β1 on L-428 cells of 0.25 nM and 0.19 nM, respectively (**Figure 1B**). In separate experiments, MHG-8 (hIgG4, mouse clone mimicking ABBV-151-like antibody) showed cellular binding within a similar EC50 of 0.22 nM (**Supplementary Figure 2C**). As platelets also express GARP/TGF-β1 on their surface, we evaluated binding of PBA-0111 to human platelets enriched from buffy coats, defined as CD45^-^CD41^+^CD42b^+^. PBA-0111 showed minimal binding (1.2-fold, ΔMFI 37 ± 14) to platelets from six individual donors (n=6) compared with afucosylated isotype control, whereas ABBV-151-like and DS-1055a-like antibodies bound 4.6- and 3.1-fold (ΔMean Fluorescence Intensity (ΔMFI) 416 ± 250 and 246 ± 127, respectively) compared to isotype control (**Figure 1C**). The minimal binding of PBA-0111 to platelets may partly reflect its lower affinity and faster dissociation from GARP/TGF-β1, as observed in our binding studies (see affinity data below), or differences in the accessibility of a structure-dependent epitope on platelet-associated GARP/TGF-β1.

**Figure 1:**
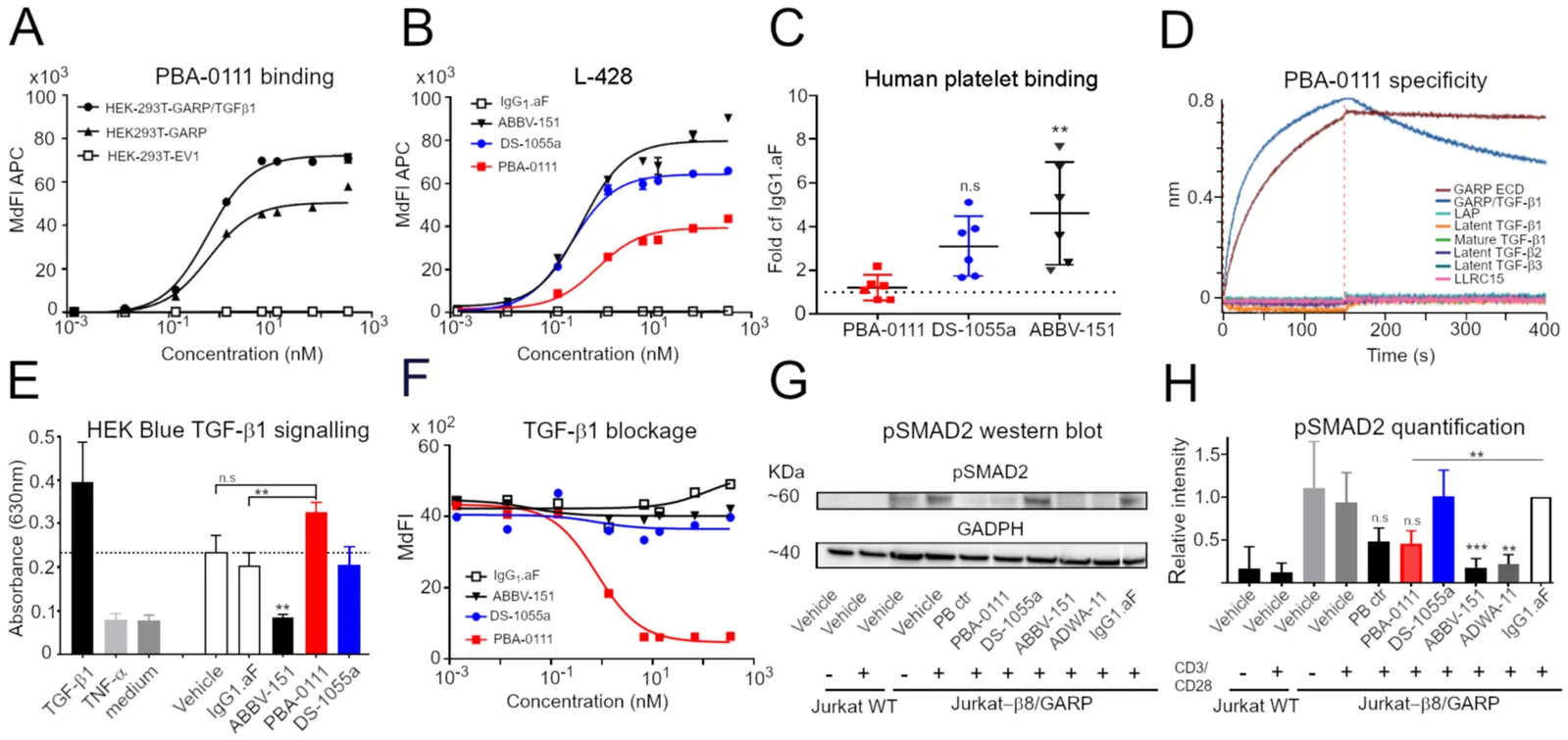
Binding properties of PBA-0111 to GARP and GARP/TGF-β1. **(A)** Increasing concentrations of PBA-0111 were titrated on HEK293T cells overexpressing GARP, GARP/TGF-β1 or empty vector (EV) control, and the binding potency was analyzed by flow cytometry. **(B)** Increasing concentrations of PBA-0111, an ABBV-151-like antibody, a DS-1055a-like antibody, or an afucosylated isotype control were titrated on L-428 lymphoma cell line, which endogenously expresses GARP/TGF-β1 and the binding potency was analyzed by flow cytometry. Data in **(A)** and **(B)** are shown as mean ± standard deviation (SD) and are representative of at least three independent experiments performed in duplicates. **(C)** Flow cytometry analysis of binding of PBA-0111, DS-1055a-like, or ABBV-151-like antibody to human platelets at 10 µg/mL. Data are expressed as fold change in median fluorescence intensity (MdFI) relative to the afucosylated isotype control, (n=6 independent donors, **p=0.0062, n.s, p=0.06 comparing to PBA-0111, Kruskal-Wallis test). **(D)** Biolayer interferometry (BLI) binding analysis of PBA-0111 against recombinant extracellular GARP, GARP/TGF-β1, latency associated peptide (LAP), TGF-β1, latent TGF-β1, latent TGF-β2, latent TGF-β3, or GARP paralog leucine-rich repeat-containing protein 15 (LRRC15) at 500 nM. All samples were tested in at least three independent experiments in duplicate. **(E)** HEK-Blue™ TGF-β reporter assay. Raji GARP/TGF-β1 cells were pre-incubated with the indicated antibodies for 30 minutes and then co-cultured with HEK-Blue™ TGF-β cells. After 48h at 37 °C, SEAP activity was assessed using QUANTI-Blue™ Solution and optical density (OD) measured at 630 nm. Data shown as mean ± SD, n= 9, from 3 independent experiments. Statistical differences were assessed by Kruskal-Wallis test with Dunn´s multiple comparisons versus vehicle or afucosylated isotype control, **p<0.001. **(F)** Flow cytometry analysis of GARP/TGF-β1 complex formation on Raji-GARP cells. Cells were preincubated with 0.2 ng/mL to 50 µg/mL of PBA-0111, ABBV-151-like antibody, DS-1055a-like antibody, or afucosylated isotype control prior to addition of recombinant latent TGF-β1 at final concentration of 1.5 µg/mL. Complex formation was detected using an anti-LAP antibody. The graph is representative of at least three independent experiments. **(G)** TGF-β1 signalling was assessed in a Jurkat cell line overexpressing β8-GARP, stimulated with plate-bound anti-CD3 beads and soluble anti-CD28 in the presence of indicated antibodies (20 μg/mL) for 24 h at 37 °C. Cell lysates were analyzed by western blot and densitometric analysis of pSMAD2 bands was performed. **(H)** pSMAD2 band intensities from WB were normalized to the loading control (GAPDH) and then to isotype control,(n= 6 gels, ABBV-151 n=8 samples, 4 MHG-8 and 4 LGH.10.6). Statistical analysis was performed using the Kruskal-Wallis test with Dunn’s multiple comparisons to IgG1.aF control, ***p=0.0002 (ABBV-151, n=8), **p=0.0037 (ADWA11). Statistical significance of PBA-0111 in single comparison to IgG1. aF was performed with Mann-Whitney analysis, p=0.002.

**Table 1:** Antibody binding properties to recombinant proteins or transfected HEK293T cells.

Sample	GARP					GARP/TGF-β1			
	K_D_ (nM)	k_on_ (1/Ms)	k_off_ (1/s)	EC50 (nM)	K_D_ (nM)		k_on_ (1/Ms)	k_off_ (1/s)	EC50 (nM)
PBA-0111	9.0 ± 3	(5.2 ± 0.5) × 10^4^	(5 ± 2) × 10^-4^	0.74	292 ± 11		(3.4 ± 0.01) × 10^4^	(1.00 ± 0.04) × 10^-2^	0.60
DS-1055a	6.0 ± 2	(2.3 ± 0.9) × 10^5^	(1.2 ± 0.2) × 10^-3^	0.35	7.0 ± 0.9		(2.2 ± 0.1) × 10^5^	(1.5 ± 0.3) × 10^-3^	0.42
ABBV-151	n. d	n. d	n. d	n. d	4.22 ± 0.3		(1.3 ± 0.01) × 10^5^	(5.7 ± 0.7) × 10^-4^	0.48

n.d, not detectable.

Surface plasmon resonance (Biacore 8K) was used to measure molecular binding affinities to GARP and GARP/TGF-β1. PBA-0111 bound the full-length extracellular domain of GARP with an affinity of 9 nM (n= 3 independent experiments, **Supplementary Figure 1C**; **Table 1**), compared to DS-1055a-like antibody with an affinity of 6 nM (**Table 1**; **Supplementary Figure 1G**), whereas ABBV-151-like antibody showed no binding, as expected (**Supplementary Figure 1E**). All three antibodies (PBA-0111, DS-1055a-like and ABBV-151-like) bound recombinant GARP/TGF-β1 complex with affinities of 292, 7 and 4 nM, respectively (**Table 1**; **Supplementary Figures 1D, F** and **H**, respectively). Similar range affinity was confirmed to MHG-8 antibody measured in Biolayer Interferometry (BLI, 2 nM; **Supplementary Figure 2B**). PBA-0111 seemed to demonstrate faster off rate from the recombinant GARP-complex, compared to recombinant GARP alone.

Biolayer Interferometry (BLI) confirmed the specificity of PBA-0111 against GARP and GARP/TGF-β1 as no binding was observed against recombinant Latent Associated Peptide (LAP), latent TGF-β1 or mature TGF-β1 (n=3). Additionally, no detectable binding was observed with GARP paralog leucine-rich repeat containing 15 (LRCC15), TGF-β2 or TGF-β3 (**Figure 1D**).

To further investigate the binding epitope of PBA-0111, a competitive BLI assay was performed using GARP, GARP/TGF-β1, PBA-0111, ABBV-151-like and DS-1055a-like antibodies. Results indicated that DS-1055a-like antibody binds a distinct epitope from ABBV-151-like antibody and PBA-0111, whereas PBA-0111 competes with ABBV-151-like antibody. This suggests that PBA-0111 and ABBV-151-like antibody recognize partly overlapping, but not identical, epitopes on GARP. Alternatively, the respective epitopes may be spatially close enough for the antibodies to sterically hinder each other’s binding (**Supplementary Figure 2A**).

### PBA-0111 blocks GARP/TGF-β1 complex formation and TGF-β1 downstream signaling

The immunosuppressive activity of regulatory T cells (Tregs) in the tumor microenvironment is partly driven by high surface expression of GARP/TGF-β1 complexes on Tregs and tumor cells, promoting TGF-β1 activation and downstream immunosuppressive signaling (12).

To assess whether PBA-0111 can block TGF-β1 release from Raji GARP/TGF-β1 cells, a TGF-β1 reporter assay was performed using the HEK-Blue™ TGF-β cell line and the QUANTI-Blue™ detection system. Raji-GARP/TGF-β1 cells were pre-incubated with PBA-0111, DS-1055a-like antibody or ABBV-151-like antibody or afucosylated isotype antibody (20 µg/mL) for 30 minutes, followed by coculture with HEK-Blue™ TGF-β cells for 48 hours at 37 °C. Secreted embryonic alkaline phosphatase (SEAP) reporter activity was then quantified spectrophotometrically as a readout of TGF-β1 signaling. In this assay, PBA-0111 did not inhibit TGF-β1 signaling and thus indirectly the release of TGF-β1 from Raji-GARP/TGF-β1 cells, as expected from its binding pattern to both free GARP and GARP-complex (**Figure 1E**). Instead, a slight increase in SEAP signal was observed compared with the no-antibody control (dashed line). This increase was not statistically significant when compared to no-antibody control pool (n=6 from three independent experiments) but was statistically significant when compared to afucosylated isotype control (p=0.0124, Kruskal Wallis test). The DS-1055a-like antibody did not affect TGF-β1 signaling whereas the ABBV-151-like antibody LHG10.6 inhibited TGF-β1 signaling in HEK-Blue™ TGF-β cells down to medium control level, consistent with its mechanism of action in blocking the release of active TGF-β1. Similar results were obtained with MHG-8-like antibody (**Supplementary Figure 2D**). TNF-α (negative control, dotted line) and medium alone yielded signal levels comparable to those observed with Raji WT controls (**Supplementary Figure 2D**), while recombinant TGF-β1 (0.025 ng/mL) served as a positive control. It should be noted, however, that in these assays, to our surprise, the reporter system did not seem to distinguish between active and latent recombinant TGF-β1, nor GARP-bound forms. Thus, the result may also reflect the antibody ability to block shedding or to block shed LTGF-β1 or GARP/TGF-β1 complexes from the Raji cells to bind and engage the reporter cells (**Supplementary Figure 2E**), and one should be careful interpreting the results. Nevertheless, PBA-0111 did not seem to either block TGF-β1 activation from pre-formed GARP/TGF-β1 complex or block binding any of the components to the reporter cells.

To determine whether PBA-0111 rather blocks the formation of GARP/TGF-β1 complex on the surface of GARP-expressing cells, Raji-GARP cells were incubated with an afucosylated isotype control antibody, PBA-0111, ABBV-151-like antibody or DS-1055a-like antibody. Antibodies were added either prior to the addition of latent TGF-β1 (LTGF-β1) or simultaneously with LTGF-β1. Complex formation was assessed by flow cytometry using fluorescently labeled anti-LAP (Latent Associated Peptide) antibody, which detects latent TGF-β1 in complex with GARP. Under these conditions, PBA-0111 inhibited *de novo* complex formation on the cell surface of GARP-expressing cells nearly 100%, with an EC50 of around 1.6 nM (**Figure 1F**). In contrast, neither the isotype control nor ABBV-151-like antibody (which does not bind GARP alone) nor the non-blocking DS-1055a-like antibody showed inhibitory activity. To exclude the possibility that the observed inhibition was due to steric hindrance with anti-LAP antibody binding, competition ELISA and flow cytometry analyses were performed, confirming that PBA-0111 does not seem to interfere with anti-LAP binding to the GARP/TGF-β1 complex (**Supplementary Figure 2F**). The blocking effect of PBA-0111 in this assay could also explain the slight increase of HEK-Blue™ signaling by PBA-0111 treatment seen in the previous assay. If PBA-0111 competes with LTGF-β1 binding to GARP, free LTGF-β1 could have accumulated in the medium increasing the HEK-Blue™ assay signal.

To further evaluate whether PBA-0111 inhibits downstream TGF-β1 signaling, Jurkat cells overexpressing GARP and the beta-8 subunit of αVβ8 integrin were used, as reported previously (22). CD3/CD28 stimulated Jurkat-β8/GARP cells were treated with PBA-0111, an afucosylated isotype control antibody, DS-1055a-like antibody, ABBV-151-like antibody LHG10.6 at 20 µg/mL for 24 hours. An internal control antibody (PB Ctr) and ADWA11 (anti-β8) were added as positive controls. Phosphorylated SMAD2 (pSMAD2), a key intracellular mediator of TGF-β1 signaling, was analyzed by western blot as a functional readout. Treatment with PBA-0111 decreased the pSMAD2 band intensities by an average of 55% (n=6 gels, p=0.002 Mann-Whitney) compared to medium only, isotype control (IgG1.aF) or DS-1055a-like antibody, albeit with a lower extent compared to ABBV-151-like antibodies LHG10.6 and MHG-8 (n=4 + 4), that decreased pSMAD intensity by average of 85% (p=0.0002, Kruskal-Wallis) compared to negative controls), (**Figures 1G, H**). Similar findings were found both for IgG1 and IgG4 formats of the MHG-8-like antibody (**Supplementary Figure 10A**). These findings suggest that TGF-β1 signaling can be inhibited either by blocking the release of active TGF-β1 from the GARP complex, as demonstrated by ABBV-151-like antibody, or by preventing formation of the GARP/TGF-β1 complex, as demonstrated by the PBA-0111 antibody.

### PBA-0111 induces ADCC against GARP positive tumor cells and Tregs and activates NK cells

PBA-0111 has been specifically designed to induce antibody-dependent cellular cytotoxicity (ADCC) against tumor cells and immunosuppressive regulatory T cells expressing GARP or the GARP/TGF-β1 complex. Its afucosylated Fc domain enhances binding to FcγRIIIA, promoting ADCC-mediated killing of GARP/TGF-β1-expressing Tregs and tumor cells by Natural Killer (NK) cells as well as FcγRIIIA-expressing non-classical monocytes and macrophages.

To demonstrate that afucosylation of the Fc domain of PBA-0111 indeed translates into enhanced ADCC, compared with PBA-0111 in natural IgG1 format, NK cells derived from healthy blood-donors were incubated with Raji cells stably transfected to express human GARP (Raji-GARP) or GARP-TGF-β1 complex (Raji-GARP/TGF-β1). Cells were incubated for 4 hours with increasing concentrations of either afucosylated or native form of PBA-0111. Afucosylated PBA-0111 induced ADCC towards Raji-GARP and Raji-GARP/TGF-β1 with a donor-averaged mean EC50 of 24 pM and 22 pM, respectively (n=6 measurements from 3 independent donors; **Supplementary Figures 3A, B**). Incubating cells with increasing concentrations of a native (i.e. fucosylated) form of PBA-0111 induced ADCC towards Raji-GARP- and Raji-GARP/TGF-β1 with an average EC50 of 254 pM and 271 pM, respectively (**Supplementary Figures 3A, B**). Thus, afucosylated PBA-0111 increased NK-mediated ADCC 10-12-fold over native antibody *in vitro*, consistent with its increased affinity for FcγRIIIA receptor. Specifically, afucosylated PBA-0111 showed affinities of 9.7 nM and 61 nM for the FcγRIIIA 158V and 158F variants, representing 12.3- and 10-fold increased affinity over the native IgG1 format, respectively (**Table 2**). For comparison, DS-1055a-like antibody, an afucosylated IgG1, displayed an affinity of 10.5 nM and 72 nM for the FcγRIIIA 158V and 158F variants, respectively.

**Table 2:** Fc binding affinities to FcγRIII variants.

Sample​	FcγRIIIA (176V)​		FcγRIIIA (176V)	
	Affinity *K*_D_ (nM)	Fold difference in *K*_D_	Affinity *K*_D_ (nM)	Fold difference in *K*_D_
PBA-0111	9.70 ± 0.04	12.3	61 ± 1	10
PBA-0111 wt	119 ± 6		665 ± 13	
DS-1055a	10.5 ± 0.4	n. a	72 ± 3	n. a

n.a., not applicable.

To test how the GARP and GARP/TGF-β1 expression levels on target cells affect PBA-0111-mediated ADCC, NK-based cytotoxicity assays were performed using target cells expressing various levels of GARP and GARP/TGF-β1, including Raji-GARP/TGF-β1, L-428 and maturated regulatory T cells derived from human peripheral blood mononuclear cells (PBMCs). The ADCC induction potential of PBA-0111 was compared to DS-1055a-like antibody. First, the capability of PBA-0111 to induce NK cell-mediated ADCC towards Raji cells overexpressing human GARP (Raji-GARP) or GARP/TGF-β1 complex (Raji-GARP/TGF-β1) was assessed using flow cytometry. In the assay, using healthy donor PBMC-derived NK cells as effector cells, after 4h incubation, PBA-0111 demonstrated a potent concentration-dependent ADCC towards Raji-GARP cells with a donor-averaged mean EC50 of 39 pM and towards Raji-GARP/TGF-β1 cells (95-145K antigens/cell), with an average EC50 of around 29 pM (**Figures 2A, B**). This ADCC induction was slightly lower than what was observed by DS-1055a-like antibody with an average EC50 of 7 pM with both cell lines. PBA-0111 did not induce ADCC towards wild-type Raji cells, a cell line negative for the target expression (**Supplementary Figure 3C**). Of note, MHG-8 in IgG1 format, in contrast to IgG4 format, also demonstrated ability to induce ADCC against Raji-GARP/TGF-β1 cells (**Supplementary Figure 10B**).

**Figure 2:**
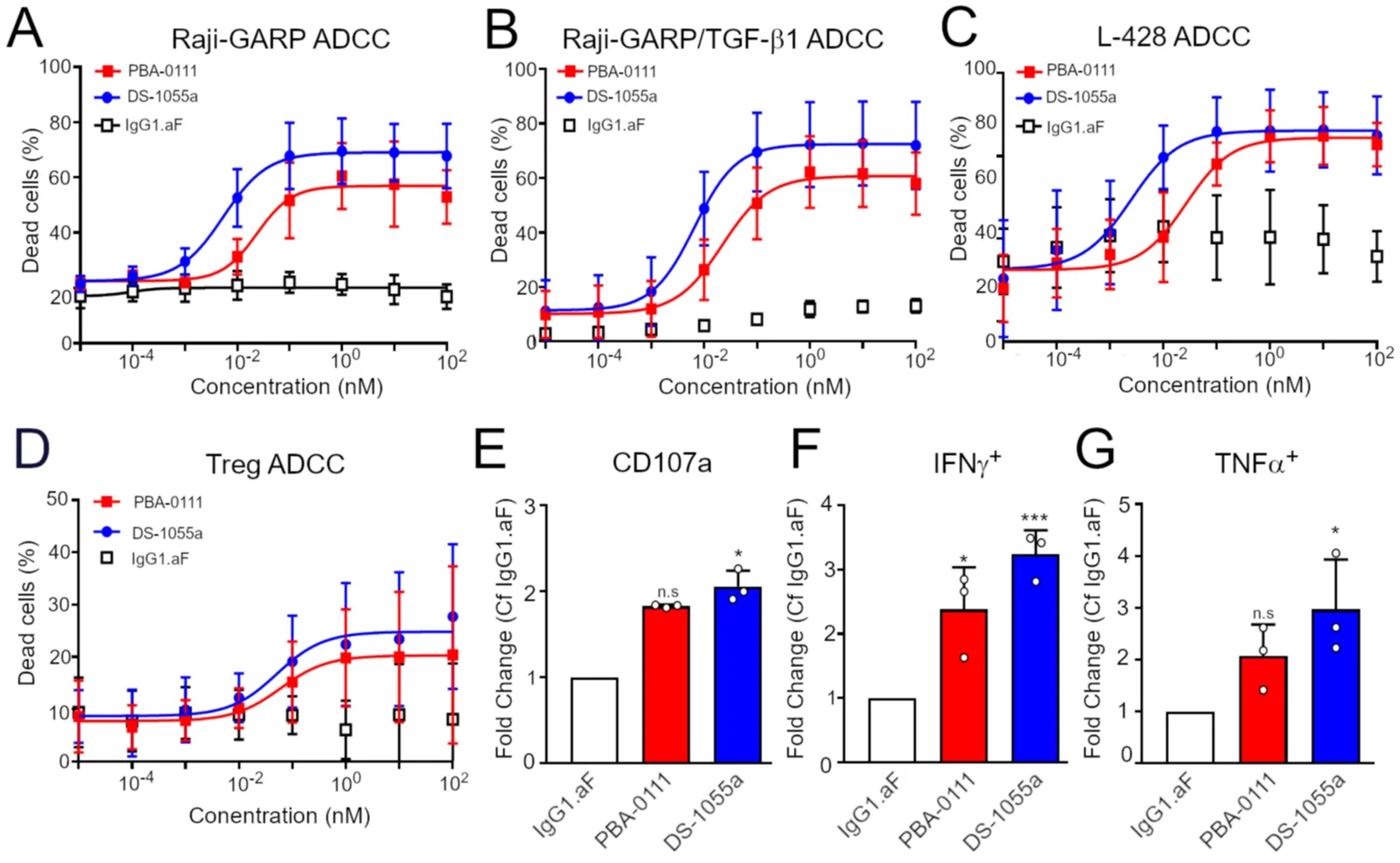
PBA-0111 demonstrates potent ADCC against GARP and GARP/TGF-β1 positive tumor cells and Tregs, accompanied by NK cell activation. **(A)** Raji-GARP target cells were treated with serial dilutions (0.00001-100 nM) of PBA-0111, DS-1055a-like antibody, or afucosylated isotype control and subjected to NK cell-mediated lysis for 4 h at 37 °C at an Effector: Target ratio of 10:1. Cell killing was analyzed by flow cytometry using live/dead stain. Data are shown as mean ± SD from a representative pool of three independent donors tested in duplicate. **(B)** ADCC was performed against Raji-GARP/TGF-β1 target cells (n=6, from 3 independent donors) using the same conditions as in **(A)**. **(C)** ADCC against the L-428 lymphoma cell line (n=6, from 3 independent donors). **(D)** ADCC against *in vitro* differentiated Tregs. CD4+CD25high T cells isolated from healthy donor PBMCs were stimulated with anti-CD3/CD28 beads to generate regulatory T cells, then subjected to autologous NK-mediated cellular lysis overnight at 37 °C in the presence of indicated anti-GARP antibodies. Data represent pooled results from three independent donors tested in duplicate, mean ± SD. **(E)** PBMCs (n=3 donors, in 2 replicates) were co-incubated with Raji-GARP/TGF-β1 cells and either PBA-0111, DS-1055a-like antibody, or afucosylated isotype control (IgG.aF) at 5 nM for 4 hours. NK cells were then analyzed by flow cytometry for CD107a expression in the presence of Golgi stop. **(F)** The same NK cells were analyzed for IFN-γ and **(G)** TNF-α production. Data are expressed as fold change relative to the afucosylated isotype control. Statistical significance versus IgG1.aF was assessed by Kruskal-Wallis with Dunn´s multiple comparison, *p< 0.05, **p< 0.01, ***p< 0.001.

To further investigate the capability of PBA-0111 to induce NK cell-mediated ADCC towards endogenously GARP/TGF-β1 expressing tumor cells, NK-mediated cytotoxicity assays were performed using L-428 cells (~8 K antigens/cell) as target cells. In these assays, using healthy donor PBMC-derived NK cells as effector cells, PBA-0111 induced a concentration-dependent ADCC with a donor-averaged mean EC50 of 33 pM (**Figure 2C**) and DS-1055a-like antibody with an EC50 of 3 pM (n= 6, 3 independent donors).

In addition to killing cancer cell lines, the potential of PBA-0111 to induce ADCC toward autologous regulatory T cells was determined, one of the main sources of immunosuppressive TGF-β1 in the tumor microenvironment (TME). Healthy donor PBMC-derived CD4^+^CD25^high^ T cells were isolated and stimulated with CD3 and CD28 beads to generate regulatory T cells. The maturation of regulatory T cells was assessed by flow cytometry (CD4^+^CD25^+^Foxp3^+^CD127^low^) and 30-50% GARP expression with high MdFI on Tregs was confirmed prior assay set-up. During the maturation of Tregs, isolated autologous NK cells were cultivated in the presence of IL-2. To demonstrate that PBA-0111 can trigger the killing of autologous regulatory T cells, NK cells and Tregs derived from the same donors were co-cultured in the presence or absence of various concentrations of PBA-0111 and DS-1055a-like antibody for 16 hours. Subsequently, the killing of Tregs was analyzed by flow cytometry. The data demonstrate high potency of regulatory T cells killing by PBA-0111 with an EC50 of 60 pM (**Figure 2D**). In the same assays DS-1055a-like antibody demonstrated an EC50 of 50 pM.

In experiments similar to ADCC, it was determined whether PBA-0111 induces activation markers on NK cells upon engagement. Human PBMCs were incubated with Raji GARP/TGF-β1 target cells at a ratio of 1:1 for 4 hours, in the presence of transport inhibitors Monensin and Brefeldin A, and either together with PBA-0111, afucosylated isotype control antibody or DS-1055a-like antibody at 5 nM. Subsequently, CD107a expression was analyzed on NK cells from PBMC using cell surface flow cytometry. Analyses of IFN-γ and TNF-α in NK cells were performed using intracellular staining after fixation and permeabilization, followed by flow cytometry analysis. In pooled data from 3 independent PBMC donors, PBA-0111 demonstrated a two-fold increase of granule exocytosis, as evidenced by increased levels of CD107a cell-surface expression by NK cells when compared to the IgG1.aF (**Figure 2E**). The increase of CD107a was similar to that observed with the DS-1055a-like antibody. Moreover, PBA-0111 triggered a statistically significant 2.4-fold increase in production of proinflammatory cytokine IFN-γ when compared to isotype control that was nearly comparable to DS-1055a-like antibody (**Figure 2F**). Finally, even if not statistically significant, PBA-0111 increased the TNF-α secretion at least 2-fold when compared to negative control (**Figure 2G**). The data confirmed that PBA-0111 activates NK cells leading to robust ADCC.

### Pharmacokinetic profile of PBA-0111 in mice

To ensure that PBA-0111 possesses pharmacokinetic properties suitable for *in vivo* anti-tumor efficacy studies in mice, a pharmacokinetic (PK) study was conducted in BALB/cJRj mice to determine the half-life of PBA-0111. Of note, PBA-0111 does not cross-react with mouse GARP/TGF-β1 complex (**Supplementary Figure 4**). Mice (n=3/time-point) received a single intravenous (IV) dose of PBA-0111 at 10 mg/kg, corresponding to the intended dose for subsequent *in vivo* efficacy studies, and blood samples were collected at multiple time-points. Plasma concentrations of PBA-0111 were quantified by ELISA using plate-bound GARP for capture and detection with an HRP-conjugated anti-human Fc antibody. Following a single 10 mg/kg dose the terminal half-life of PBA-0111 in BALB/cJRj mice was determined to be 4.4 days, Cmax 198 µg/mL and AUCINF 790 day*µg/mL (**Supplementary Figure 3D**), consistent with the pharmacokinetic profile expected for afucosylated antibodies.

### PBA-0111 induces anti-tumor efficacy in tumor models expressing GARP/TGF-β1 either on tumor cells or regulatory T-cells

The anti-tumor efficacy of PBA-0111 was first evaluated in a tumor model designed to mimic human tumors with GARP expression on tumor cells. Human CD34^+^ hematopoietic stem cell (huHSC) humanized NSG-hIL15 mice (n=6-7/group) were inoculated subcutaneously with Raji-GARP/TGF-β1 cells on the right flank. Once tumors reached a tumor volume of 80-150 mm^3^, mice were randomized based on huHSC donor and tumor volume. Mice were treated with intravenous injections of vehicle, DS-1055a-like antibody, or PBA-0111 at 10 mg/kg body weight, twice a week for a total of six doses. Treatment with PBA-0111 resulted in statistically significant tumor growth inhibition, with a 39% reduction in tumor volume compared to the vehicle group (p<0.0001, two-way ANOVA) at study termination (**Figure 3A**). The anti-tumor effect of PBA-0111 was greater than that observed with the reference DS-1055a-like antibody, which did not demonstrate any statistically significant anti-tumor efficacy compared to vehicle (13%, p=0.3195). The anti-tumor efficacy of PBA-0111 did not reach statistically significant difference compared to DS-1055a-like antibody in this model (p=0.108). The individual tumor growth curves are shown in **Figure 3B**. PBA-0111 was well-tolerated, with no notable clinical signs such as weight loss, behavioral changes or alterations in posture, fur texture, or skin integrity. In an independent experiment using the same tumor model MHG-8 IgG1 demonstrated statistically significant tumor growth inhibition compared to vehicle on day 17 (p=0.0022, Kruskal-Wallis test) and showed a statistically significant difference compared with MHG-8 IgG4 on day 20 (p= 0.0377, **Supplementary Figure 10C**).

**Figure 3:**
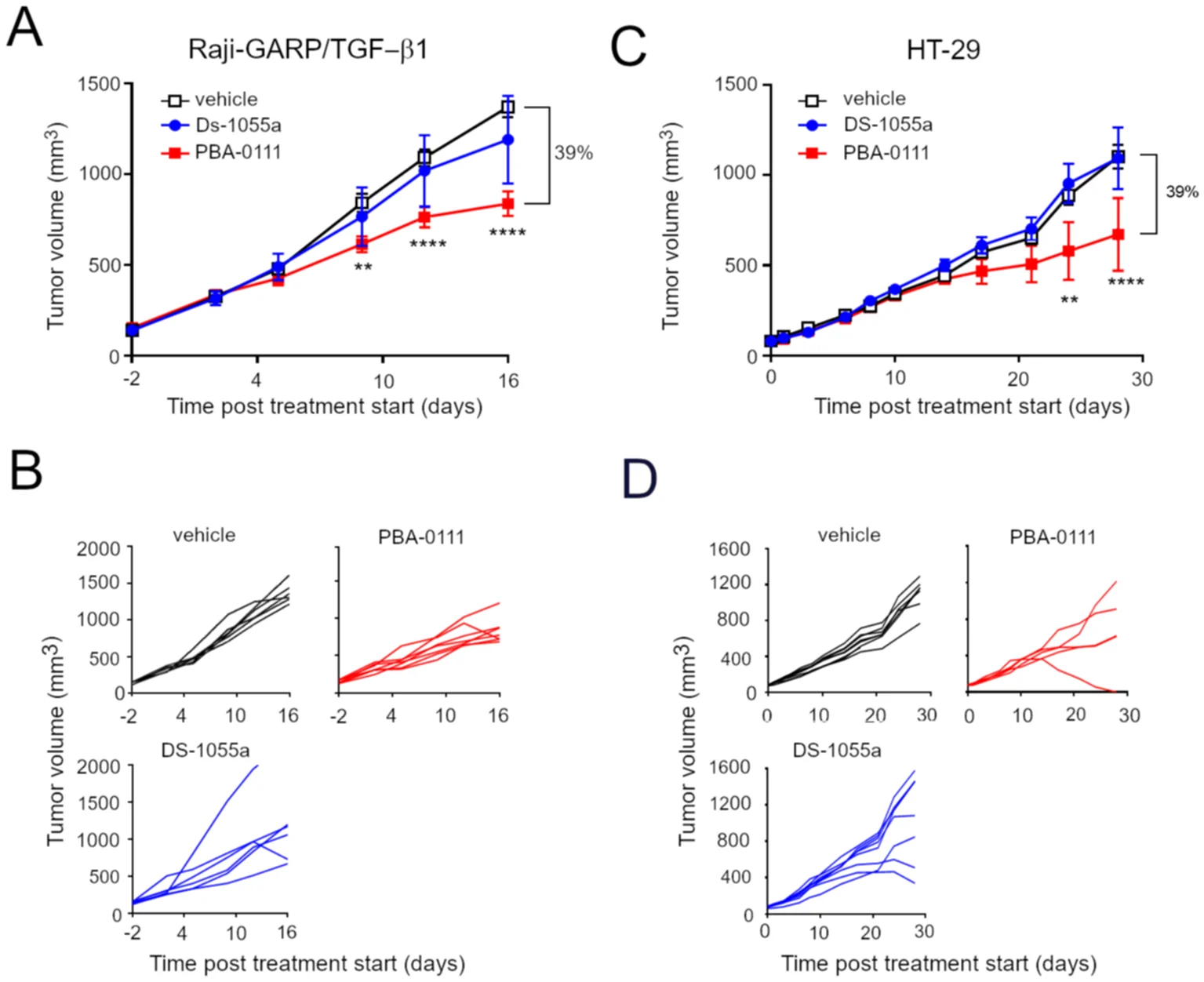
PBA-0111 induces anti-tumor efficacy in hCD34+ HSC humanized tumor models expressing Raji-GARP/TGF-β1 complex on tumor cells or regulatory T cells **(A)** CD34^+^ HSC humanized NSG-hIL15 mice were inoculated subcutaneously with Raji-GARP/TGF-β1 cells and treated with six intravenous doses of PBA-0111 or DS-1055a-like antibody at 10 mg/kg twice weekly starting when the tumor volume was around 80-150 mm^3^. Vehicle was used as a negative control. Data are shown as mean ± SEM (n=6-7). Individual tumor growth curves are shown in **(B)**. **(C)** CD34^+^ HSC humanized NCG-hIL15 mice were inoculated subcutaneously with HT-29 colon carcinoma cells and treated with six intravenous doses of PBA-0111 or DS-1055a-like antibody at 10 mg/kg twice weekly, starting when the tumor volume was around 80 mm^3^. Data are shown as mean ± SEM (n=5-8). Individual tumor growth curves are shown in **(D)**. Statistical significance was assessed using two-way ANOVA, with Bonferroni multiple comparisons, **p< 0.01, ****p< 0.0001.

To further assess anti-tumor efficacy in a model where tumor cells are GARP-negative but tumor-infiltrating activated Tregs express GARP/TGF-β1, studies were performed using human HT-29 colon carcinoma tumor-bearing CD34^+^ HSC humanized NCG-hIL15 mice. HT-29 cells were implanted subcutaneously into the right flank/shoulder, and mice (n=8/group) were randomized when tumors reached an average volume approximately 80 mm^3^, based on HSC donor, level of human immune cell reconstitution, and tumor size. Mice were treated with intravenous injections of vehicle, PBA-0111, or DS-1055a-like antibody at 10 mg/kg, administered twice a week for six doses. The tumor volumes and body weights were monitored two to three times a week. In this tumor model, PBA-0111 demonstrated statistically significant tumor growth inhibition (p< 0.0001, two-way Anova, Bonferroni’s multiple comparison to vehicle) with 39% reduction in tumor volume compared to the vehicle treatment group at the study termination (**Figures 3C, D**). Moreover, PBA-0111 demonstrated greater anti-tumor efficacy at the termination tumor volume (p=0.0015, two-way Anova, Bonferroni’s multiple comparison to DS-1055a) than the DS-1055a-like antibody, which did not show any anti-tumor efficacy in this model.

Unexpectedly, treatment with PBA-0111 in this model was associated with acute adverse effects in a subset of mice, resulting in mortality in 3 out of 8 mice. Transient clinical signs, including impaired movement, labored breathing and changes in fur appearance, were observed within 15-30 minutes following intravenous administration but were resolved within an hour in all surviving mice. These effects were not observed with subsequent dosing, and analysis of the remaining animals did not detect any abnormalities like weight loss, increased GVDH score, or cytokine release (**Supplementary Figures 5A–C**). Nor were cytokines detected in mouse bronchoalveolar lavage fluid (BALF) in subsequent experiments, even at doses up to 30 mg/kg (**Supplementary Figure 5D**). The adeverse effects were unexpected, as no cross-reactivity to mouse GARP/TGF-β1 had been detected in prior binding studies to recombinant GARP/TGF-β1 (**Supplementary Figure 4A**). Further studies indicated that the toxicity was murine-specific, as the greatest toxicity was observed in non-humanized mice (**Supplementary Table 2**). Binding analysis of PBA-0111 to isolated mouse spleen, lung and white blood cells did not demonstrate any significant binding to any of the cell types analyzed (**Supplementary Figures 4B, C**, **6**). In a follow-up experiment, these adverse effects were mitigated by stepwise intravenous dosing and were not observed by subcutaneous or intraperitoneal administrations. Additional safety assessments conducted under similar conditions, including exploratory non-GLP safety studies in hCD34^+^-NCG-hIL15 mice using stepwise dosing, showed no evidence of hematological abnormalities, changes in platelet counts, or fibrinogen levels and no histopathological changes (**Supplementary Tables 3**, **4**; **Supplementary Figures 5F, E**) discussed further in the discussion.

### PBA-0111 activates immune cells *in vivo*

To confirm the therapeutic mode-of action of PBA-0111 *in vivo*, the tumors, spleens, and blood were collected at the termination of anti-tumor experiments described above. Spleens and tumors were dissected, one half of each sample was processed into single-cell suspension, stained with markers for distinct effector cell populations and activation status, and analyzed by flow cytometry. The second half of the tumor was fixed and retained for immunohistochemistry.

In terminal samples from the Raji-GARP/TGF-β1 tumor model, flow cytometric analysis demonstrated a statistically significant increase in CD3^+^ T cells in the PBA-0111 treated tumors compared with vehicle treated tumors. These cells consisted predominantly (up to 90% by PBA-0111 treatment), of cytotoxic CD8^+^ T cells (**Figure 4A**). In contrast, the reference DS-1055a-like antibody did not affect T cell populations in this model. Although regulatory T cells (Tregs) were generally present at low levels, PBA-0111 reduced both their number and relative proportion (**Supplementary Figure 3E**). Consequently, the cytotoxic T lymphocyte (CTL)/Treg ratio increased more than five-fold compared to vehicle control (**Figure 4B**), reaching statistical significance. The DS-1055a-like antibody produced a smaller reduction in the FoxP3^+^ population and did not affect the CTL/Treg ratio.

**Figure 4:**
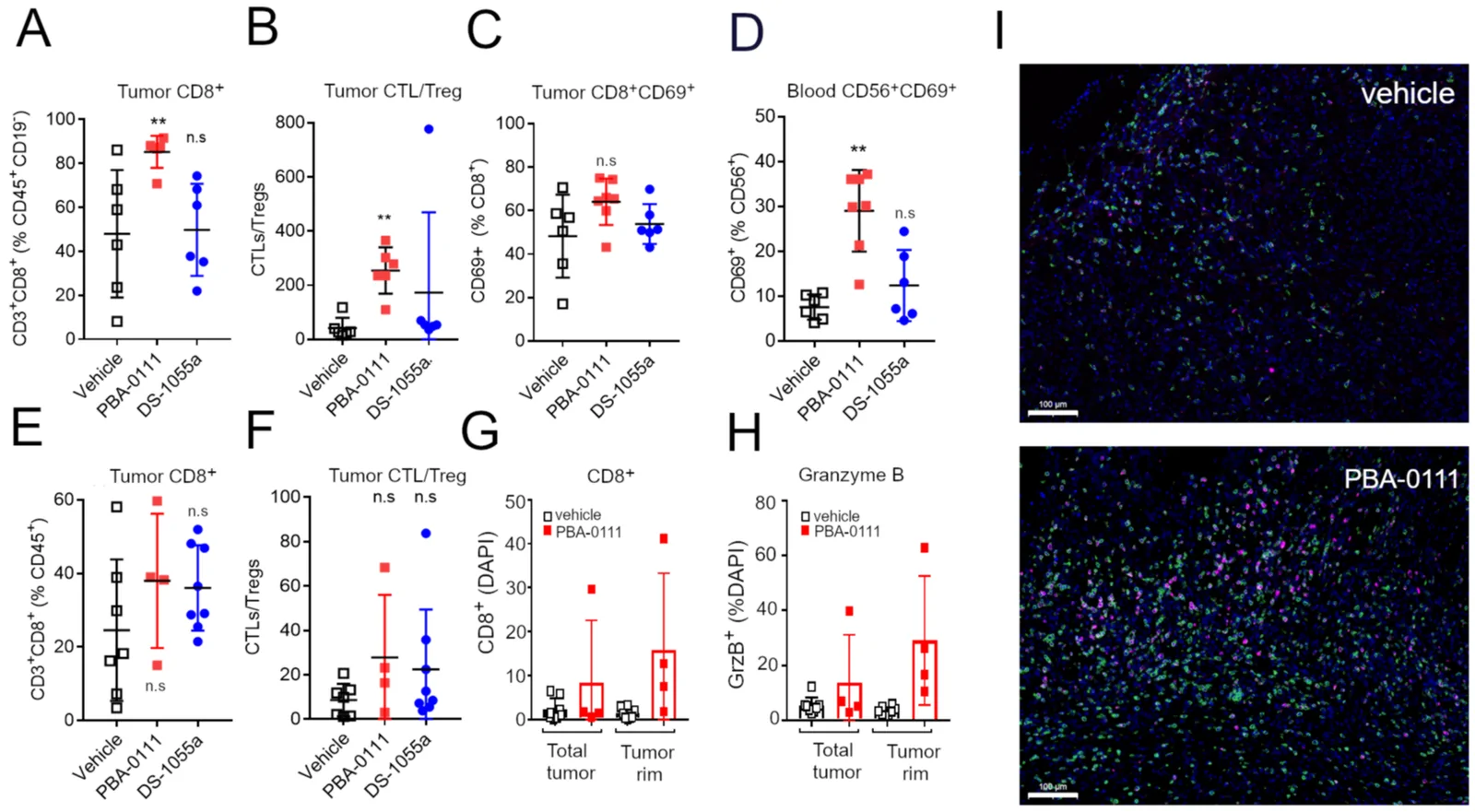
PBA-0111 activates T cells leading to anti-tumor efficacy. **(A–D)** CD34^+^ HSC humanized NSG-hIL15 mice (n=6-7/group) were inoculated subcutaneously with Raji-GARP/TGF-β1 and treated with vehicle or six doses of PBA-0111 or DS-1055a-like antibody at 10 mg/kg twice weekly, initiated when the tumor volumes reached 80-150 mm^3^. At study termination, tumors, spleens and terminal blood were collected and processed for FACS analysis. **(A)** Proportion of CD8^+^ cells within the tumor **(B)** Intratumoral CD8^+^/Treg ratio. **(C)** Activation status of intratumoral CD8^+^ T cells. **(D)** NK cell activation in peripheral blood. **(E–I)** CD34^+^ HSC humanized NCG-hIL15 mice (n=8/group) were inoculated subcutaneously with HT-29 colon carcinoma cells and treated as above. At study termination, tumors and terminal blood were processed for FACS analysis (n=4-8/group). **(E)** Proportion of intratumoral CD8^+^ T cells. **(F)** Intratumoral CD8+/Treg ratio. **(G)** CD8^+^ T cell infiltration confirmed by immunohistochemistry (IHC). **(H)** Granzyme B expression detected by IHC. **(I)** Representative IHC images showing CD8^+^ (green) and Granzyme B^+^ (magenta) cells in immune-positive clusters at the tumor periphery (rim) of HT-29 tumors. Scale bar = 100 µm. Statistical significance was assessed by Kruskal-Wallis test with Dunn´s multiple comparisons. **p < 0.01.

In addition to increasing the proportion of CD8^+^ T cells, PBA-0111 treatment showed a trend toward enhanced cytotoxic T cell activation, as indicated by an increased proportion of CD69-positive CD8^+^ T cells, although this did not reach statistical significance (**Figure 4C**). PBA-0111 did not affect the total number of NK cells within the tumors, rather the proportion among tumor infiltrating leukocytes appeared slightly reduced (**Supplementary Figure 3F**), partly reflecting the increased fraction of CD8^+^ T cells. This was in contrast to the DS-1055a-like antibody, which increased the proportion of NK cells in both tumor and spleen (**Supplementary Figures 3F, G**), although these changes were not statistically significant. Notably, in the peripheral blood, PBA-0111 treatment induced almost threefold increase in NK cell activation, measured by CD69 upregulation, which was statistically significant (**Figure 4D**). No notable effects were observed in the proportions of Tregs or NK cells in the blood (**Supplementary Figures 3H, I**).

In terminal samples from the HT-29 tumor model, flow cytometric analysis, similarly to Raji-GARP/TGF-β1 model, indicated an increase in frequency of CD8^+^ T cells in the tumors from PBA-0111 treated animals compared to vehicle-treated controls (**Figure 4E**). However, this increase was less pronounced than in the Raji-GARP/TGF-β1 model and did not reach statistical significance, potentially due to lower overall GARP expression in this tumor model. A comparable increase in CD8^+^ T cells was also observed with the DS-1055a-like antibody (**Figure 4E**). The frequency of Tregs was higher in HT-29 tumors than in the Raji-GARP/TGF-β1 model, however, there were no significant changes by either antibody treatment (**Supplementary Figure 3J**), and the CTL/Treg ratio was only modestly increased following PBA-0111 and DS-1055a-like antibody treatment (**Figure 4F**). No increases in CD56^+^ NK cells were observed in the tumor environment (**Supplementary Figure 3K**). The increased CD8 frequency in PBA-0111 treated tumors was confirmed by immunohistochemistry (**Figures 4G, I**), where CD8^+^ T cells were primarily localized in clusters at the tumor margin. Consistent with this observation, increased Granzyme B (GrzB) expression was detected in the PBA-0111-treated group, predominantly in CD8^+^ T cells within the same peripheral tumor regions (**Figures 4H, I**). No significant changes were observed in peripheral blood immune cell populations or activation markers in this model.

### Pharmacodynamic trends of PBA-0111 in soft tissue sarcoma patients

To investigate whether the preclinical data would translate into a clinical setting, we performed a Phase 0 clinical trial in patients with soft tissue sarcoma receiving intra-tumoral injections of PBA-0111 using the Comparative *In Vivo* Oncology (CIVO^®^) platform (32). CIVO^®^ technology involves intratumoral injection of multiple microdoses of drugs in a spatially discrete manner, enabling localized assessment of drug activity within the native tumor environment (**Supplementary Figure 9**) (33, 34).

Six patients received intratumoral injections of PBA-0111 at a total dose of 149.4 *μ*g, administered using an eight-needle configuration of the CIVO^®^ injector (24.9 µg/needle, 6 needles, 2 vehicles). Patients underwent pre-scheduled surgical resection 2-96 hours post injection, based on the planned tumor resection (**Figure 5A**), after which tumors were processed for immunohistochemistry analysis. All six enrolled patients had tumors with evaluable injection sites, yielding a distinct, visually trackable injection track at 47 of 48 sites. These 47 identified injections produced 128 distinct injection sites across three to four z-level sections per sample. After excluding sites affected by necrosis or located outside tumor tissue, 43 of 48 injection sites (89.6%) were analyzed, corresponding to 103 of 128 identified injection sites (80.5%) in total (**Supplementary Table 6**).

**Figure 5:**
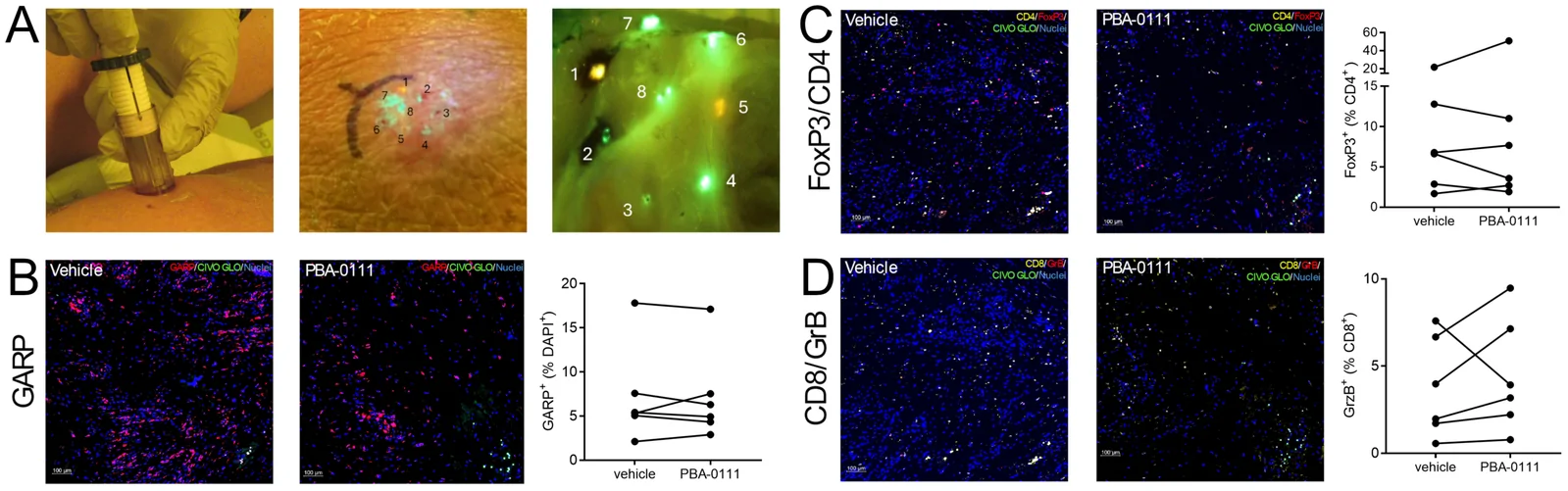
PBA-0111 shows signs of T cell activation in soft tissue sarcoma patients following intratumoral administration. **(A)** CIVO workflow. PBA-0111 was co-formulated with fluorescent microspheres and loaded into the CIVO injector, which delivers eight individual columns of drug directly into the patient tumor (left). STS patients receiving the multiplexed microinjection were evaluated immediately following the procedure by visualization of individual injection sites using a blue light and yellow filter that illuminates the CIVO GLO fluorescent microspheres with an ultraviolet flashlight (center). Following a pre-scheduled surgical resection 2-96 hours later, the tumors were sectioned transverse to the drug columns, yielding 2-4 sections of FFPE tissue for downstream IHC and ISH staining and image analysis. **(B)** GARP expression assessed by ISH. Representative injection site images from vehicle and PBA-0111-treated sites in Patient 5, with summary data showing the median percentage of GARP^+^ cells across all evaluable injection sites in all patients (n=6). **(C)** Regulatory T cell infiltration assessed by IHC. Representative injection site images for vehicle and PBA-0111-treated sites in Patient 5, with summary data showing the median percentage of CD4+ T cells that are FoxP3^+^ across all evaluable injection sites in all patients. **(D)**. CD8^+^ cytotoxic T cell activity assessed by IHC. Representative injection site images for vehicle and PBA-0111-treated sites in Patient 5, with summary data showing the median percentage of CD8^+^ T cells that are Granzyme B^+^ across all evaluable injection sites in all patients.

Two treatment-emergent adverse events (TEAEs) were reported across two of the six patients (2/6, 33.3%). The first was Grade 2 (moderate) deep vein thrombosis, for which a relationship to the CIVO^®^ device, injection procedure, and/or the injected contents were deemed possible. The second was a Grade 3 (severe) postoperative wound infection, deemed unrelated to the CIVO^®^ device or to the injected contents. No TEAEs led to study discontinuation, no injection site reactions were reported, and no fatalities occurred.

To assess tumor microenvironment (TME) responses to localized drug exposure following intratumoral microinjection, biomarker analysis was performed by immunohistochemistry (IHC) or *in situ* hybridization (ISH) against a predetermined panel encompassing GARP expression, regulatory T cells, effector cells, and markers of immune activation.

GARP transcript expression, evaluated by ISH, was detected across all patient samples, albeit at generally low levels. The proportion of GARP-positive cells varied considerably between patients, ranging from 3.2% in patient 2 to 16.7% in patient 6, with a heterogeneous distribution across patients and injection sites (**Figure 5B**; **Supplementary Table 7**). Interestingly, in some injection sites, but not in all, GARP-positive cells were found more frequently in immune cell-enriched areas co-localizing with CD4^+^FoxP3^+^ Tregs, consistent with the known preferential expression of GARP on activated Tregs.

NK cell frequency was consistently low throughout the study, with median values of 0.5-2.5% at both vehicle and PBA-0111 injection sites across all patients (**Supplementary Table 7**). Tumor cells in some patients also stained positively for CD56, complicating accurate quantification of the CD56^+^GrB^+^ cytotoxic subpopulation. No meaningful differences between vehicle and PBA-0111-treated sites could be determined for this population. FoxP3^+^ Treg frequency was similarly low across all patients, with total cell percentages remaining below 2%. Nevertheless, a modest reduction in the proportion of CD4^+^FoxP3^+^ population was observed at PBA-0111-treated sites relative to vehicle in both patient 3 (0.482% vs. 0.844%, median) and patient 5 (0.119% vs. 0.162%), suggesting a possible trend towards local Treg reduction in a subset of patients. The Foxp3^+^ in CD4^+^ population in patient 5 was reduced from 7.4% in the vehicle treated sites to 3.9% in PBA-0111 treated sites (**Figure 5C**).

The proportion of CD8^+^ T cells in the TME varied considerably across patients, ranging from 2.3% in patient 5 to 14.0% in patient 3. Despite generally low levels of Granzyme B (GrB)-expressing CD8^+^ T cells at both vehicle- and PBA-0111-treated sites, a consistent trend toward increased CD8^+^GrB^+^ cytotoxic T cell activity at PBA-0111-treated sites was observed in five of six patients (**Figure 5D**; **Supplementary Table 7**), albeit this did not reach statistical significance. The most pronounced effect was seen in patient 5, where GrB positivity among CD8^+^ T cells nearly doubled from 4.04% at vehicle sites to 7.14% at PBA-0111-treated sites, a change that coincided with the reduction in CD4^+^FoxP3^+^ Tregs described above. The highest absolute proportion of activated cytotoxic T cells at PBA-0111-treated sites was observed in patient 4, where GrB positivity reached 10.7% compared to 7.8% at vehicle sites.

No other significant changes in immune cell composition were detected. Inflammatory cytokines and chemokines, including IFN-γ, TNF-α, ISG15, and CXCL10, were not elevated, and cleaved caspase-3 did not provide evidence of detectable tumor or Treg cell killing following treatment (**Supplementary Table 7**). The limited immune changes across patients may reflect low GARP expression on tumor or Treg populations, or insufficient effector cell availability within the TME. These findings should also be interpreted in the context of the limitations of localized intra-tumoral administration, small number of patients evaluated and cofounding effects from previous treatments. Nonetheless, the increase in CD8^+^ T cell-associated activation markers at PBA-0111-treated sites, observed in this exploratory analysis, is consistent with findings from the HT-29 xenograft model and provides preliminary evidence of localized pharmacodynamic activity in a clinical setting.

## Discussion

Although TGF-β1 represents a compelling therapeutic target in cancer, global pathway inhibition carries significant on-target toxicities, supporting the development of more selective approaches such as inhibition of GARP-mediated TGF-β1 activation (7, 8). GARP is expressed on activated Tregs, platelets, endothelial cells and certain tumor cells, where it tethers latent TGF-β1 to the cell surface and facilitates its local activation. This restricted expression pattern has made GARP an attractive therapeutic target for several clinical-stage antibodies (20, 23, 26, 35).

Here we describe PBA-0111, a fully human afucosylated IgG1 that integrates two different mechanisms of action. PBA-0111 binds both GARP and GARP/TGF-β1 complex, enabling it to simultaneously induce ADCC in against GARP-positive tumor cells and Tregs (depletion), and prevent GARP/TGF-β1complex formation, thereby blocking downstream TGF-β1 signaling (blocking). *In vitro*, PBA-0111 demonstrated ADCC activity against GARP-positive tumor cells and Tregs comparable to the DS-1055a-like reference antibody. Functionally, PBA-0111 blocked TGF-β1 binding to GARP and significantly suppressed pSMAD2 phosphorylation, achieving similar pathway inhibition to ABBV-151-like antibody, but through a distinct mechanistic entry point, through prevention of complex formation rather than blockade of TGF-β1 activation within a pre-formed complex. The biological relevance of targeting GARP/latent TGF-β1 complex formation is supported by the observation that GARP is expressed on the Treg surface independently of TGF-β1 loading, generating a population of unoccupied GARP molecules available to capture extracellular latent TGF-β1 (36, 37). *In vitro* studies have demonstrated that free surface GARP can bind exogenous latent TGF-β1, suggesting this route may contribute to the immunosuppressive TGF-β1 pool on Tregs (37, 38). Furthermore, PIIO-1 the GARP/TGF-β1 complex blocking antibody described by Li et al, demonstrated anti-tumor efficacy *in vivo*, both as a murine antibody and humanized IgG1. This tumor growth reduction was accompanied by reduced intratumoral Treg frequencies, decreased pSMAD3 activation, and increased frequencies of CD8^+^ cytotoxic T cells exhibiting activated phenotype (20). Thus, PIIO-1 antibody data indicates that blocking complex formation *in vivo* is a viable alternative pathway to inhibit TGF-β1 activation.

Our *in vivo* studies, using both GARP/TGF-β1-complex positive tumor cells and tumors with GARP expression restricted to Tregs, also demonstrated tumor growth inhibition accompanied by increased frequency of cytotoxic T cells. In the Raji-GARP/TGF-β1 tumor model, in which the GARP/TGF-β1 complex is overexpressed on tumor cells, PBA-0111 produced robust tumor growth inhibition accompanied by a significant increase in intratumoral cytotoxic CD8^+^ T cells, consistent with findings reported for PIIO-1 (20). By contrast, DS-1055a, which induces ADCC but does not affect TGF-β1 activation, produced a different immune profile, characterized by increased NK cell activation rather than T cell infiltration. This distinction likely reflects mechanistic differences between the two antibodies. In this model, GARP/TGF-β1 expressed by tumor cells may function as a key immunosuppressive signal that PBA-0111 relieves by indirectly inhibiting the TGF-β1 signaling, allowing CD8^+^ T cell expansion. DS-1055a, lacking this blocking capacity, may instead primarily engage CD16-expressing NK cells through ADCC without sufficiently countering the immunosuppressive TGF-β1 signal. It is also plausible that PBA-0111, in contrast to DS-1055a, was not only able to kill GARP positive Tregs and tumors cells but also modulated Treg survival and conversion of conventional CD4^+^ T cells to Tregs by suppressing TGF-β1 activation (39). This could potentially explain the observed differences in Treg counts/proportions between PBA-0111 and DS-1055a-like treated tumors, despite DS-1055a-like antibody demonstrating slightly more potent cytotoxic activity *in vitro* compared to PBA-0111.

It has been described that the ABBV-151-like anti-mouse GARP antibody 58A2 failed to induce any significant anti-tumor efficacy, nor did it increase cytotoxic T cell proportions in the CT26 and MC38 tumor models without PD-1 combination therapy (15, 35). Therefore, we performed a separate validation experiment using ABBV-151-like MHG-8 antibody in the humanized mice with Raji-GARP/TGF-β1 tumor, both as a human IgG1 (depleting activity combined with TGF-β1 activation blocking) and IgG4 (TGF-β1 activation blocking). Both formats produced meaningful tumor growth inhibition accompanied by CD8^+^ T cell expansion (**Supplementary Figure 10**). MHG-8 as human IgG4 format (clinically developed format) inhibited the tumor growth by 34%, and the MHG-8 hIgG1 format showed somewhat greater inhibition (53%), consistent with the known contribution of Fc-mediated effector function to anti-tumor activity and further supporting the rationale for the afucosylated IgG1 format of PBA-0111 and dual-functioning antibodies. Furthermore, the IgG1 format seemed to activate CD8^+^ T cells more potently compared to IgG4, in terms of slightly increased CD69 positivity. While this comparison was made with MHG-8 rather than PBA-0111, and in a separate donor cohort, it provides indirect mechanistic support that Fc-effector function contributes activity beyond TGF-β1 blockade alone, consistent with, though not proof of, the rationale for combining both mechanisms in PBA-0111.

The Raji-GARP/TGF-β1 model may also serve as a surrogate for GARP/TGF-β1-mediated immune suppression in the tumor microenvironment, where tumor cells expressing GARP/TGF-β1 functionally mimic Treg-like suppression of T cells. Lucas and colleagues recently published that GARP-mediated TGF-β1 suppression of T cells operates over short distances, with pSMAD2^+^ Tumor infiltrating lymohocytes (TILs) found preferentially within approximately 70 µm of FoxP3^+^ Tregs (40). Thus, normally only a fraction of tumor-infiltrating T cells would fall within the suppressive radius at any given time. In the Raji model, however, this uniform and abundant GARP/TGF-β1 surface expression would place virtually every infiltrating CD8^+^ T cell within suppressive range simultaneously. Relief of this uniformly distributed suppression therefore can produce broader CD8^+^ T cell responses compared to models with more restricted GARP/TGF-β1 expression. While the Raji-GARP/TGF-β1 model represents an artificial tumor context due to forced overexpression of the complex, this feature also makes it a useful tool for mechanistically dissecting the contribution of tumor-expressed GARP/TGF-β1 to immune suppression *in vivo*.

In the HT-29 colorectal tumor model, where endogenous GARP is expressed predominantly on Tregs rather than tumor cells, both PBA-0111 and DS-1055a demonstrated a trend toward increased cytotoxic T cell frequency, consistent with Treg-mediated activity in a model with slightly higher Treg abundance. However, PBA-0111 again showed better anti-tumor efficacy compared to DS-1055-like antibody, which may reflect its additional capacity to block TGF-β1 activation in a Treg-rich environment, compounding the effect of Treg depletion. Notably, the proportion of Tregs was not significantly affected in this model, which may be explained by conversion of conventional CD4 T cells to Tregs, relatively low GARP expression on Tregs as observed by flow cytometry or insufficient local density of ADCC effector cells. Immunohistochemical analysis confirmed the increased CD8^+^ T cell infiltration into the tumor parenchyma following PBA-0111 treatment, along with elevated Granzyme B expression, without a significant increase in cleaved caspase-3 in Foxp3^+^ cells, suggesting that direct Treg killing may not be the sole driver of the observed anti-tumor effect in this model. Whether this reflects an intrinsic limit on ADCC efficacy at low, Treg-restricted target density, limited effector cell availability despite the afucosylated Fc of both antibodies, or continuous Treg replenishment via TGF-β1-driven CD4^+^ conversion remains to be resolved in future studies.

The lack of significant anti-tumor activity for DS-1055a-like antibody in this CD34^+^ HSC-humanized model contrasts with its previously reported, modest activity in a huPBMC-humanized model (26). Humanized mouse platforms differ in immune reconstitution kinetics, effector repertoire, Treg number, and GvHD susceptibility, and these model- and donor-dependent factors likely contribute to this discrepancy (41–43). The low Treg number and GARP expression discussed above, together with the later dosing start and less frequent dosing schedule used in this experiment, may further explain the reduced efficacy of DS-1055a-like antibody.

A notable and unexpected finding in HT-29-tumor-bearing humanized NCG-hIL15 mice was the occurrence of sudden deaths in a subset of treated animals, of which some presented with lung edema upon necropsy. Thorough investigation failed to identify a definitive cause, but several mechanistic possibilities were systematically excluded. A possible LPS contamination or antibody aggregation was promptly disqualified. Cross-reactivity against LRRC15, a structural paralog to human GARP, was absent, and PBA-0111 showed no binding to latent TGF-β1, 2, or 3. Binding studies by Microscale Thermophoresis confirmed no cross-reactivity of PBA-0111 with recombinant mouse or rat GARP/TGF-β1 complex, and FACS analysis of mouse tissues, including Tregs and platelets, revealed no detectable PBA-0111 binding, in contrast to an anti-mouse GARP antibody. Critically, analysis of blood and bronchoalveolar lavage from affected animals showed no evidence of cytokine release, and blood biochemistry, hematology, and fibrin levels were within biological variation, effectively excluding platelet dysfunction as a contributing mechanism. Moreover, TGF-β pathway-related toxicities typically manifests as chronic rather than acute pathology, making a TGF-β-mediated mechanism unlikely (44). Histopathological examination of all major organs, including lungs and heart, showed no treatment-related changes.

The mouse-specific nature of this finding was further supported by subsequent studies across multiple immunocompetent (BALB/c, C57BL/6) and immunodeficient (NCG-IL15 or NCG-SGM3) strains, both humanized (hCD34) and non-humanized. Adverse effects were most pronounced in non-humanized mice and were slightly attenuated in animals engrafted with human hematopoietic cells. This pattern is consistent with the hypothesis that human GARP expression competes with a yet unidentified mouse target for PBA-0111 binding, potentially redirecting the antibody away from the putative off-target. The Raji-GARP/TGF-β1 xenograft model, in which abundant human GARP is present, produced no adverse events, further supporting this interpretation. No notable adverse events were observed in rats, and alternative dosing routes (subcutaneous, intraperitoneal) or stepwise intravenous administration mitigated the effect in mice that was only seen during the first dosing. As the precise molecular target responsible for these seemingly mouse-specific events has not yet been identified, it underscores the importance of comprehensive cross-reactivity profiling against a full spectrum of potential off-targets during further preclinical development, and future preclinical safety investigations are planned before entering phase 1 clinical trials.

Analysis of tumor-infiltrating lymphocytes from six soft tissue sarcoma patients treated with intratumoral administration of PBA-0111 (NCT06700070) provided an exploratory clinical assessment of immune pharmacodynamic findings observed in the HT-29 model. No significant changes in immune cell composition were observed, nor were elevations in inflammatory cytokines or chemokines, including IFN-γ, TNF-α, ISG15, or CXCL10, detected. Similarly, cleaved caspase-3 did not provide evidence of increased tumor-cell or Treg apoptosis following treatment. The lack of significant changes in cell composition, immune cells activation or cell death in these patients can potentially reflect low GARP expression on tumor or Treg populations or limited local effector cell availability.

The patient GARP expression analysis was performed by using ISH, based on slight non-specific staining observed with anti-GARP antibodies in our HT-29 xenograft tumors that were paraffin-embedded and in the initial histological screenings of patient tumor samples. This specificity issue with anti-GARP antibodies has also been observed by others (40). Since GARP expression was assessed by ISH, these measurements may not accurately reflect GARP protein levels or subcellular location. Indeed, it has been demonstrated previously that GARP mRNA expression may correlate poorly with protein levels (40, 45). These technical limitations likely contributed to the apparent uniformity of GARP expression across the six patients despite differences in immune response. One patient showed notably distinct patterns of Treg frequency and CD8^+^ T cell activation compared to the others, which may partly reflect inter-patient variability in GARP protein expression not captured by ISH, differential activation status of Tregs and thus GARP expression, differences in effector cell composition, but also the spatially restricted nature of GARP/TGF-β1 immunosuppression (26, 37, 38). The variability in the spatial proximity between GARP-expressing Tregs, TGF-β1, and cytotoxic T cells across patients may therefore contribute to differential responsiveness to PBA-0111 treatment (40). Furthermore, prior radiotherapy and/or anti-PD-1 therapy (wash period 26-56 days) may also have influenced the baseline tumor immune microenvironment, representing an important confounder for these exploratory pharmacodynamic findings in a limited number of patients. Timing of tumor resection relative to treatment also varied across patients, depending on surgical scheduling. Notably, Patient 6’s tumor was resected approximately 2 h post-administration, likely too early to capture meaningful changes in adaptive immune-cell composition or activation, representing a further limitation. Nevertheless, trends toward decreased Treg frequency in a subset of patients and elevated Granzyme B expression in cytotoxic T cells in five of six patients were consistent with the pattern of T cell activation observed in the immunodeficient humanized mouse models, supporting the translational relevance of the preclinical data. Future clinical trials should consider incorporating patient stratification based on GARP protein expression and intratumoral effector cell composition.

In summary, PBA-0111 is a fully human afucosylated antibody that represents a differentiated approach to GARP-targeted therapy, combining ADCC-mediated depletion of immunosuppressive GARP-expressing cells with prevention of local TGF-β1 activation in the tumor microenvironment. Anti-tumor efficacy was demonstrated in models in which GARP is expressed on tumor cells as well as in models where GARP expression is restricted to Tregs, with both settings generating robust anti-tumor responses accompanied by increased cytotoxic T cell activation. In soft tissue sarcoma patients treated with intratumoral PBA-0111, exploratory analyses showed preliminary signs of local immune activation, including T cell engagement, consistent with preclinical findings. Together, these mechanistic, preclinical and early clinical observations presented here support PBA-0111 as a rationally designed dual-mechanism antibody warranting further clinical investigation.

## Data Availability

The original contributions presented in the study are included in the article/**Supplementary Material**, further inquiries can be directed to the corresponding author/s.
