# Supplementary material for: A fully human afucosylated anti-GARP antibody PBA-0111 drives anti-tumor immunity through combined ADCC induction and TGF-β1 pathway blockade
Source: Front Immunol. 2026 Sep 17;17:1921265. doi: 10.3389/fimmu.2026.1921265 (PMC13627347; doi:10.3389/fimmu.2026.1921265)
Supplement: Supplementary file 1 [file SupplementaryFile1.docx]

Supplementary Material

A fully human afucosylated anti-GARP antibody PBA-0111 drives anti-tumor immunity through combined ADCC induction and TGF-β1 pathway blockade

**Pieter Spee^1^, Monika Litwin^1^, Damian Trojanowski^1^, Connor Burns^2^, Adrianna Dudek^1^, Elżbieta Gołdyn^1^, Kamilla Sołtys^1^, Michał Sobecki^1^, Aleksandra Świderska^1^, Irwin Matyjaszczyk^1^, Marek Orłowski^1,3^, Piotr Mamczur^1^, Robert Kołodziejczyk^1^, Dmytro Piliuhin^1^, Jianmin Xu^4^, Ewelina Krzywińska^1^, Sameer Deshmukh^1^, Tomasz Klaus**^1^**, Ewelina Świderek**^1^**, Kate Gillespie^2^, Atticus Turner^2^, Wendy Jenkins^2^, Jason Frazier^2^, Kenneth R Gundle^5^, John Weinberg^1^, Niina Veitonmäki^1*^**

# 1 Supplementary Material and Methods

## 1.1 Epitope binning

Epitope competition was evaluated using the Octet RED384 (Sartorius). The tested antibodies at 5 µg/mL were immobilized on sensors, followed by binding of GARP/TGF-β1 complex (1$\mu$M) and subsequent association of secondary antibody-GARP/TGF-β1 complex premixes (0.5 µM, and 1 µM respectively). Blocking responses were normalized to self-blocking controls.

## 1.2 MicroScale Thermophoresis analysis

MicroScale Thermophoresis (MST) analysis was performed using fluorescently labelled PBA-0111-AF647 at a final concentration of 1 nM against cynomolgus monkey GARP/TGF-β1/His (Acro Biosystems, #GA1-C52W), mouse GARP/TGF-β1/His (Acro Biosystems, #GAI-M52W2), and rat GARP/TGF-β1/His-Avi (Kactus, #GAT-RM401) at concentrations ranging from 0.5 to 1000 nM. Measurements were performed using a Monolith NT.115 Pico instrument (NanoTemper, #MO-K025) in premium capillaries with HBS-N buffer supplemented with 0.04% NP-40. Instrument settings were as follows: MST power, High; LED power, 5%; temperature, 25°C. Data were analysed using MO Affinity Analysis v2.3 (NanoTemper). MST traces were evaluated using the following time intervals: cold region start, −2.45 s; cold region end, −1.45 s; hot region start, 0.1 s; and hot region end, 2.1 s. KD values were determined by steady-state analysis from three independent measurements.

## 1.3  Anti-LAP ELISA

StrepTactinXT-coated plates (IBA Lifesciences, 2-4101-001) were coated with recombinant GARP/TGF-β1 (Pure Biologics) at 2 µg/mL and incubated overnight at 4°C. The following day washed plates were blocked with 2% Bovines serum albumin for one hour at RT. PBA-0111 samples were generated using serial dilutions of PBA-0111 in 2% BSA/PBS solution and incubated on the plate for one hour followed by washes. Anti-LAP-AF647 (R&D systems, IC77541R) were added in either 10x or 100X EC50 excess for another hour, followed by anti-mouse IgG HRP conjugated (R&D systems, HAF007) and TMB substrate (BD OptEIA™ ). Sample absorbance was read in plate reader at 450 nm and 650 nm (reference) wavelengths.

## 1.4 Pharmacokinetics in BALB/cJRj mice

A pharmacokinetic study was conducted in nine female BALB/cJRj mice by Trulylabs (Lund, Sweden). Following intravenous administration of PBA-0111 at 10mg/kg, blood samples were collected via sublingual bleeding at 1min, 3 h, 6 h, 24 h, 48 h,72 h,7 days, and 14 days post-dose. At the terminal point, mice were anesthetized with 3-4% isoflurane, and final blood collection was performed via cardiac puncture, followed by euthanasia by cervical dislocation. A serial sampling design was employed, in which each animal contributed samples at three time points, with three animals bled per time point. An additional three mice received vehicle and served as controls. Prior to dosing, all animals were weighed and individually identified by ear marking. Blood samples were processed to obtain plasma according to standard procedures, and plasma samples were frozen at -80ºC until analysis.

Plasma concentrations of PBA-0111 were determined using an enzyme-linked immunosorbent assay (ELISA) specific for binding to the GARP/TGF-β1 complex. Briefly, 96-well Strep-Tactin^®^XT microplates (IBA Lifesciences, 2-4101-001) were coated with GARP/TGF-β1 (Pure Biologics) at 2 µg/mL and incubated overnight at 4°C. The following day washed plates were blocked with 2% Bovines serum albumin for one hour at RT. Plasma samples were diluted serially in 2% BSA and 2% mouse serum in PBS. Standard curves were generated using serial dilutions of PBA-0111 and MHG-8 was included as positive control at 5 µg/mL. Bound antibodies were detected using goat anti-human F(ab')2 cross-adsorbed HRP detection antibody (Invitrogen, 31414). Signal detection was performed with tetramethylbenzidine (TMB ) substrate (reagents A and B mixed 1:1, BD Pharmingen, 555214). The reaction was stopped by addition of 1M H_2_SO_4_ and absorbance was measured at 450 nm with a reference wavelength of 650 nm using a microplate reader. Pharmacokinetic parameters (e.g., Cmax, t_1/2_, AUC) were calculated using non-compartmental analysis with WinNonlin™ software.

## 1.5 Flow cytometry analysis of PBA-0111 binding to mouse tissue

Blood, spleen and lungs were collected from BalB/c mice and prepared for flow cytometry. Isolated cells were stained for live/dead eflour506, CD45-BV650. mCD31-BV605. mCD3-FITC. mCD4-APC-H7, mCDDF-PE-CF594, mCD25-PE-cy7, mFoxP3-ef450, mGARP-PE. mCD11b-perc-cy5.5, mLY6G-AF700, and either PBA-0111 of IgG1.aF with APC Goat anti-human IgG Fcγ Antibody (cat:109-605-098). Platelets were analyzed separately using mCD61-FITC.

## 1.6 Exploratory non-GLP safety study

Sixty-eight (68) females and surplus huHSC-NCG-hIL15 mice at age of 14.1-14.4 weeks were subcutaneously injected with HT29 cells resuspended in DPBS. The huHSC-NCG-hIL15 model was generated by reconstitution of CD34+ huHSC from 5 independent donors in irradiated (60 cGy, for 63 seconds) female NCG-hIL15 mice. The immune reconstitution level was confirmed by flow cytometry (mCD45, hCD45, hCD3, hCD19, hCD56). The humanization of the mouse model was identified as hCD45+/live single >20% in the peripheral blood before the inoculation of tumor cells.

When the average tumor size reached about 153 mm3 the mice were randomly assigned to groups. HuHSC-NCG-hIL15 tumor bearing mice from different huHSC donors were evenly distributed into each experimental group, and each group contained mice from all the huHSC donors. The mice in groups received stepwise intravenous administration of PBA-0111 at 0.006/0.06/0.615, 0.02/0.2/2 and 0.06/0.615/6.15 mg/kg, respectively at time 0h/4hrs/24hrs.Dosing of the mice was performed as stepwise dosing, and the dosing volume of PBA-0111 was adjusted based on body weight (5 μL/g). The mice were monitored for moribundity and mortality daily. Mice body weight and GvHD score (clinically observed) were measured 2-3 times per week. Tumor volumes were evaluated 3 times per week after grouping. Ten mice of each group were terminated 24 hours after the final dosing (termination 1). Five mice of each group were terminated on Day 15 (termination 2, 2 weeks after the last administration). Two mice per group were surplus animals for unexpected, non-treatment related events. At necropsy, an assessment for body weight. GvHD score, macroscopic abnormalities, standard hematology, clinical chemistry, fibrinogen levels, and histopathological assessment based on Hematoxylin/Eosin staining of heart, lung, brain, liver, kidney, spleen, colon was performed. Histopathology assessment was performed in blinded manner.

## 1.7  Patient Material Immunohistochemistry

Tissue sections were immunohistochemically stained with antibodies to detect effects on both cancer and immune cells. Sections were baked for 1 hour at 60ºC, deparaffinized in Clear-Rite 3, and rehydrated via graded alcohols. Sections underwent antigen retrieval for 20 minutes at 100ºC, followed by a 3% peroxidase block for 10 minutes, and one hour block in 5% normal goat serum. Sections were incubated overnight in primary antibody. A list of primary antibodies used is included in Methods Table 1. Next, the sections were incubated for one hour in AlexaFluor secondary antibody or horseradish peroxidase (HRP) secondary antibody for detection using Opal Tyramide Signal Amplification (TSA) fluorophores (Akoya) as specified in Methods Table 2. Slides were counterstained with DAPI for 10 minutes and coverslipped with Prolong Gold mounting medium (Invitrogen). Whole slide images were acquired at 20x magnification using a Zeiss AxioScan 7 (Zeiss).

**Methods Table 1. Patient IHC primary antibody information.**

**Methods Table 2. Patient IHC secondary antibody information.**

## 1.8 In situ hybridization

In situ hybridization was completed using the RNAscope multiplex fluorescent reagent kit v2 (Advanced Cell Diagnostics). Slides were baked for 1 hour at 60ºC , deparaffinized in Clear-Rite 3, and rehydrated via graded alcohols. Hydrogen peroxide was added for 10 minutes to quench endogenous peroxidase activity. Slides underwent a 15-minute target retrieval solution incubation at 100ºC , followed by a 15-minute protease digestion at room temperature. The RNAscope ISH assays were completed with probes from Advanced Cell Diagnostics listed in Methods Table 3 and Opal TSA detection (Akoya). The slides were counterstained with DAPI for 10 minutes and coverslipped with Prolong Gold mounting medium (Invitrogen). Whole slide images were acquired at 20x magnification using a Zeiss AxioScan 7 (Zeiss).

**Methods Table 3.Probe information**

# 2 Supplementary Figures and Tables

## 2.1 Supplementary Tables

**Supplementary Table 1 Comparison of anti-GARP antibodies**

**Supplementary Table 2 Comparison of mouse species for safety assessment**

**Supplementary Table 3 Histopathology analysis and inflammatory scores of exploratory non-GLP safety assessment**

*Histopathological assessment of abnormalities, performed blindly, were mainly associated with human immune infiltration in mouse tissues. Spleens showed white pulp atrophy and a decrease in the number of lymphocytes, and extramedullary hematopoiesis typical for the model. Lungs demonstrated inflammatory cells infiltration. One vehicle brain demonstrated minor inflammatory cell infiltration and a small amount of glial cell hyperplasia. In the liver tissues, minor inflammatory infiltration in hepatic sinusoids was observed related to the experimental model used. Slight fibrous tissue hyperplasia was observed in one mouse of vehicle (1/10), and two in PBA-0111 group (2/10). One mouse in vehicle showed degenerative necrosis in bile duct epithelial cells, and one in PBA-0111 showed multifocal hepatocellular necrosis. N/A, not applicable. The table shows PBA-0111 using a stepwise dosing regimen to reach cumulative total doses of 6.8 mg/kg

**Supplementary Table 4 Clinical chemistry analysis of exploratory non-GLP safety assessment**

The table shows PBA-0111 using a stepwise dosing regimen to reach cumulative total doses of 0.68 (low), 2.2 (middle) or 6.8 mg/kg (high). ALT, alanine aminotransferase, AST, aspartate aminotransferase, BUN, blood urea nitrogen, GLU, glucose, CK, creatine kinase

**Supplementary Table 5. Patient characteristics**

**Supplementary Table 6. Number of sites analyzed**

*Total number of vehicle and PBA-0111

**Supplementary Table 7. Patient immunological characteristics**

## TX= Treatment, RT= Radiotherapy, Pembro= Pembrolizumab, LLRC32= GARP, CC3= Cleaved caspase-3. Colors are based on % change vehicle- PBA-0111 / vehicle. Red indicates increase, blue decrease. Wash-out period 26-56 days.

## Supplementary Figures

**Supplementary Figure 1. Binding properties of GARP antibodies.** (A) Different concentrations of ABBV-151-like antibody were titrated on HEK293T overexpressing GARP, GARP/TGF-β1 or empty vector (EV) control cell line and the binding of ABBV-151-like antibody was analyzed by flow cytometry. (B) Binding of DS-1055a-like antibody to transfected cell lines. (C) SPR binding analysis of PBA-0111 antibody to recombinant extracellular GARP at doses between 23.4-750 nM. (D) SPR binding analysis of PBA-0111 antibody to recombinant extracellular GARP/TGF-β1 at doses between 39,1-2500 nM. (E) SPR binding analysis of ABBV-151-like antibody to recombinant extracellular GARP at doses between 5-500 nM. (F) ABBV-151-like antibody to recombinant extracellular GARP/TGF-β1 at doses between 7.81-500 nM. (G) SPR bindinganalysis of DS1055a-like antibody to recombinant extracellular GARP at doses between7.81-250 nM. (H) SPR binding analysis of DS1055a-like antibody to recombinant extracellular GARP/TGF-β1 at doses between 7.81-500 nM. All graphs are an exemplary experiment of minimally three experiments performed in duplicates or triplicates. Fitted curves are shown in black. The cellular binding experiment graphs show mean ± standard deviation (SD).

**Supplementary Figure 2 Characterization and validation of GARP/TGF-β1-targeting antibodies and assay validation** (A) Epitope binning analysis by biolayer interferometry (BLI). Epitope competition was evaluated using the Octet RED384 instrument (Sartorius). The tested antibodies at 5 µg/mL were immobilized onto sensors by amine coupling, followed by binding of the GARP/TGF-β1 complex (1$\mu$M) and subsequent association of secondary antibody-GARP/TGF-β1 complex premixes (0.5 µM, and 1 µM respectively). Blocking responses were normalized to self-blocking controls (B) Binding affinity of MHG-8 IgG4 to the recombinant human GARP/TGF-β1 complex was determined by biolayer interferometry (BLI). (C) cellular binding of MHG-8 IgG1, DS-1055a-like antibody, control IgG1 and an afucosylated isotype control. Increasing concentrations of the antibodies were titrated on L-428 lymphoma cell line. Data are presented as mean ± standard deviation (SD) and are presentative of three independent experiments performed in duplicate.(D) HEK Blue TGF-β1 signalling assay performed as described in Figure 1, comparing ABBV-151-like antibodies MHG-8 and LGH.10.6, (n= 9 replicates from three independent experiments). Statistical analysis was performed using the Kruskal-Wallis test with comparison to the IgG1.aF control, *, p=0.0261, ****, p<0.001. No statistically significant difference was observed between MHG-8 and LHG.10.6 (p= 0.2957). (E) Activity of recombinant TGF-β1, LTGF-β1, and GARP/TGF-β1 complex in HEKBlue TGF-β signalling assay. Each protein was tested at 10pg (final concentration of 0.14 ng/mL, n= 4 replicates. The experiment was repeated twice. (F) Assessment of potential interference by PBA-0111 in the anti-LAP ELISA. StrepTactinXT plates were coated overnight at 4° with recombinant GARP/TGF-β1 complexes and subsequently blocked with 2% BSA in PBS. Serial dilutions of PBA-0111 (0.002-10000ng/mL) were added to the plates and incubated for 1 h. Following washings, anti-LAP antibody was added at 100-fold excess relative to EC50 binding and incubated for 1 h. Bound antibody was detected using goat anti-human F(ab')2-HRP and TMB substrate.

**Supplementary Figure 3. *In vitro* NK-mediated cytotoxicity, pharmacokinetic profile, and *in vivo* immune cell modulation by afucosylated PBA-0111** (A) Raji-GARP target cells were treated with serial dilutions (100-0.00001 nM) of native- and afucosylated formats of PBA-0111 and subjected to NK-mediated cellular lysis for 4 h at 37°C. Cell killing was analyzed by flow cytometry in the presence of live/dead stain. (B) Raji-GARPT/TGF-β1 target cell killing in the similar settings as in A. (C) Raji-EV cells as target cells were treated at 100 nM of either afucosylated isotype control, PBA-0111 or DS-1055a-like antibody and the NK mediated cellular lysis was analyzed by flow cytometry. The graphs A-C are a representative donor of at least three individual donors tested with duplicate samples. (D) Pharmacokinetic profile of PBA-0111 in BALB/cJRj mice after single intravenous injection at 10 mg/kg (n=3/time point). (E-I) Tumors, spleens and blood from huHSC-NSG-hIL15 mice inoculated with Raji-GARPT/TGF-β1 tumor cells and treated with vehicle, PBA-0111 or DS-1055a-like antibody at 10 mg/kg were analyzed by flow cytometry at the termination, n=6-7. (E) Tumor Treg analysis by FoxP3^+^ marker (F) Tumor NK cell analysis by CD3^-^CD56^+^ markers, (G) Spleen NK cell analysis by CD3^-^CD56^+^ markers, (H) Blood cytotoxic T cells analysis by CD45^+^CD3^+^CD8^+^ markers. (I) Blood Treg analysis by FoxP3^+^ marker. n.s = not significant, *, p<0.05, Kruskal-Wallis with Dunn´s multiple comparison test compared to vehicle. Tumors from huHSC-NCG-hIL15 mice inoculated with HT-29 colon carcinoma cells and treated with vehicle, PBA-0111 or DS-1055a-like antibody at 10 mg/kg were analyzed by flow cytometry at the termination, n=4-8/group. (J) Tumor Treg analysis by CD4_+_FoxP3^+^ CD25^+^ marker. (K) Tumor NK cell analysis by CD3^-^CD56^+^ markers, No statistically significant differences were observed compared to vehicle (Kruskal-Wallis test).

**Supplementary Figure 4. Species-specific binding profile of PBA-0111 to rodent GARP/TGF-β1 and mouse immune cells.** (A) MicroScale Thermophoresis (MST) analysis of fluorescently labelled PBA-0111-AF647 (1 nM) binding to cynomolgus monkey GARP/TGF-β1/His, mouse GARP/TGF-β1/His, and rat GARP/TGF-β1/His-Avi (0.5–1000 nM) using a Monolith NT.115 Pico instrument (NanoTemper). Data represent three independent experiments (n = 3). (B) Flow cytometric analysis of PBA-0111 binding to Treg cells among splenocytes from BALB/c mice (n = 3 mice). An anti-mouse GARP antibody was used as a positive control, and an isotype-matched antibodies were used to assess background staining. (C) PBA-0111 showed no detectable binding to mouse platelets, identified as CD61⁺ cells. Gating was based on the isotype control IgG1.aF (n = 3 mice).

Supplementary Figure 5 Safety assessment of PBA-0111 in huHSC-NCG-IL15 mice. HT-29 tumor bearing mice were treated twice a week with indicated antibodies at 10 mg/kg and assessed for safety by (A) body weights and B) graft-versus-host disease GvHD score (weight, posture, fur texture and skin integrity). Graphs show mean ± SEM. NB, PBA-0111 shows the remaining animals. No statistically significance differences were observed for either parameter measured compared to vehicle. (Kruskal-Wallis test, n=5-8/group). (C) Six hours post-treatment, mice were bled retro-orbitally, and plasma was analyzed by flow cytometry for human cytokines using the BD Cytometric Bead Array (CBA) Human Th1/Th2 Cytokine Kit II. (D) To exclude local cytokine release in the lungs, huHSN-NCG-IL15 mice were treated with up to 30 mg/kg of PBA-0111, and 30 minutes post-treatment, bronchoalveolar lavage (BAL) was performed. BAL fluid (BALF) was assessed for human cytokines by flow cytometry using the CBA kit, n=3-5. The experiment was further repeated at a lower dose of 10 mg/kg. (E, F) HT-29-bearing huHSC-NCG-IL15 mice underwent non-GLP safety assessment following intravenous administration of PBA-0111 using a stepwise dosing regimen to reach cumulative total doses of 0.68, 2.2, or 6.8 mg/kg. Ten animals per dose were euthanized at 24 h (n = 10/dose) and five animals per dose at two weeks (n = 5/dose). Tissues and blood were analyzed for standard safety parameters. (E) Platelet counts at the 24 h timepoint (n = 10/group). (F) Fibrinogen levels at the 24 h and 2-week timepoints (n = 10 and n = 5/group, respectively). No statistically significance differences were observed for any of the parameters measured compared to vehicle. (Kruskal-Wallis test, or Mann-Whitney test).

Supplementary Figure 6 PBA-0111 binding to isolated lung cells from BALB/c mice. Lungs were collected from BALB/c mice (n = 3) and processed for flow cytometry. Isolated cells were stained with cell-type markers and either an anti-murine GARP antibody, IgG1 isotype control, or PBA-0111. Graph shows binding to lung-derived cells, anti-mGARP was a control in both panels.

**Supplementary Figure 7. Gating strategy for NK cell activation *in vitro.***

**Supplementary Figure 8. Gating strategy for tumors, spleens and blood from *in vivo* anti-tumor efficacy studies.**

**Supplemental Figure 9. CIVO Phase 0 Clinical Trial.** (A) The Comparative *In Vivo* Oncology (CIVO^®^) Platform enables simultaneous testing of multiple drug candidates directly in living tumors in cancer patients. Up to eight drugs or drug combinations are microinjected at spatially discrete sites within the tumor, 2-96 hours prior planned surgical resection. Each injection site is tracked using co-injected fluorescent microspheres (CIVO GLO), maintaining spatial separation of injected drugs. Post-resection, tumors can be analyzed via multiplex immunohistochemistry, in situ hybridization, and spatial omics across multiple tissue (z) levels, enabling assessment of local pharmacodynamic responses including apoptosis, proliferation, DNA damage, interferon signaling, and immune modulation. (B) After fluorescent IHC/ISH staining and scanning, each injection site is mapped using concentric rings of 100 μm width, extending from the injection center out to 1500 μm. The innermost 200 μm are excluded to avoid wound-response artefacts. Within each ring, cells are identified by DAPI staining and classified as biomarker positive or negative based on signal intensity. Ring-level data are then averaged across patients and injected drugs to generate radial plots showing the percentage of positive cells at each 100 μm interval from the injection site. These radial plots are further condensed into box plots representing the average percentage of positive cells across the full 200-1500 μm injection area, summarized per injected drug

**Supplementary Figure 10 Characterization of MHG-8 IgG1 and IgG4 antibodies and anti-tumor efficacy associated with increased intratumoral CD8⁺ T cells.** (A) Quantification of pSMAD_2_ western blot. CD3/CD28 stimulated Jurkat-β8/GARP cells were treated with MHG-8 IgG1, MHG-8 IgG4, ADWA11-like (anti-αvβ8) or IG1.aF antibodies, as described in Figure 1, and pSMAD2 expression quantified using Western blotting. Data presents four independent gels that were not included in Figure 1 (n=4). Statistical analysis was performed using Kruskal-Wallis test with Dunn’s multiple comparison to the IgG1.aF control, *, p=0.0352 (MHG-8 IgG4), p=0.0284 (MHG-8 IgG1), and p=0.015 (ADWA11-like). (B) Raji-GARP/TGF-β1 target cells were treated with 100 nM of MHG-8 IgG1 or IgG4 and subjected to IL-15 overnight stimulated NK cell-mediated lysis for 4 h at 37°C (pooled data, n= 7 replicates from two independent donors). Target cell killing was quantified by flow cytometry using live/dead stain. The dashed line indicates NK cell background. Statistical significance between the IgG1 and IgG4 formats was assessed using Mann-Whitney test (p= 0.011). (C) CD34^+^ HSC humanized NSG-hIL15 mice (n= 8/group, two HCS donors) were inoculated subcutaneously with Raji-GARP/TGF-β1 cells and treated with six intravenous doses of MGH-8 IgG1 or MHG-8 IgG4-like antibodies (10 mg/kg twice weekly) starting when the mean tumor volume reached approximately 125 mm^3^. The vehicle-treated mice was served as the negative control. Data are shown at days 17 and 20 post treatment start, mean ± SD (n=4-8). At study termination, tumors, spleens and terminal blood were collected and processed for FACS analysis. (D) Proportion of CD8^+^ T cells within the tumor (E) Activation status of intratumoral CD8^+^ T cells. For panels C-E statistical significance was assessed using the Kruskal-Wallis test with Dunn’s multiple comparisons test, with all groups compared to one another. *p < 0.01,**p < 0.01, ***p < 0.01.
